# Ultra Wideband Indoor Positioning Technologies: Analysis and Recent Advances †

**DOI:** 10.3390/s16050707

**Published:** 2016-05-16

**Authors:** Abdulrahman Alarifi, AbdulMalik Al-Salman, Mansour Alsaleh, Ahmad Alnafessah, Suheer Al-Hadhrami, Mai A. Al-Ammar, Hend S. Al-Khalifa

**Affiliations:** 1 Computer Research Institute, King Abdulaziz City for Science and Technology, Riyadh 11442, Saudi Arabia; maalsaleh@kacst.edu.sa (M.A.); aalnafessah@kacst.edu.sa (A.A.); 2 Computer Science Department, King Saud University, Riyadh 11451, Saudi Arabia; salman@ksu.edu.sa (A.A.-S.); 430204094@student.ksu.edu.sa (S.A.-H.); 3 Department of Computer Science, Al-Imam Mohammad bin Saud Islamic University, Riyadh 11432, Saudi Arabia; mai.alammar@ccis.imamu.edu.sa; 4 Information Technology Department, King Saud University, Riyadh 11451, Saudi Arabia; hendk@ksu.edu.sa

**Keywords:** Ultra Wideband, UWB, localization, positioning, indoor positioning, wireless sensor networks, wearable computing, SWOT

## Abstract

In recent years, indoor positioning has emerged as a critical function in many end-user applications; including military, civilian, disaster relief and peacekeeping missions. In comparison with outdoor environments, sensing location information in indoor environments requires a higher precision and is a more challenging task in part because various objects reflect and disperse signals. Ultra WideBand (UWB) is an emerging technology in the field of indoor positioning that has shown better performance compared to others. In order to set the stage for this work, we provide a survey of the state-of-the-art technologies in indoor positioning, followed by a detailed comparative analysis of UWB positioning technologies. We also provide an analysis of strengths, weaknesses, opportunities, and threats (SWOT) to analyze the present state of UWB positioning technologies. While SWOT is not a quantitative approach, it helps in assessing the real status and in revealing the potential of UWB positioning to effectively address the indoor positioning problem. Unlike previous studies, this paper presents new taxonomies, reviews some major recent advances, and argues for further exploration by the research community of this challenging problem space.

## 1. Introduction

Positioning is the process of determining positions of people, equipment, and other objects. It has recently been an active research area in which much of the research focuses on utilizing existing technologies to address the problem of positions’ determination. Positioning can be classified into two types, depending on the environment in which the positioning is conducted: outdoor positioning and indoor positioning. Whereas outdoor positioning is performed outside buildings, indoor positioning is performed inside buildings (e.g., houses, hospitals, and malls). Different applications may require different types of positioning technologies that fit their needs and constraints. For example, Global Positioning System (GPS) is a technology that is suitable and efficient for outdoor spaces rather than indoor spaces because satellite radio signals cannot penetrate solid walls and obstacles [1,2,3,4].

**Figure 1:**
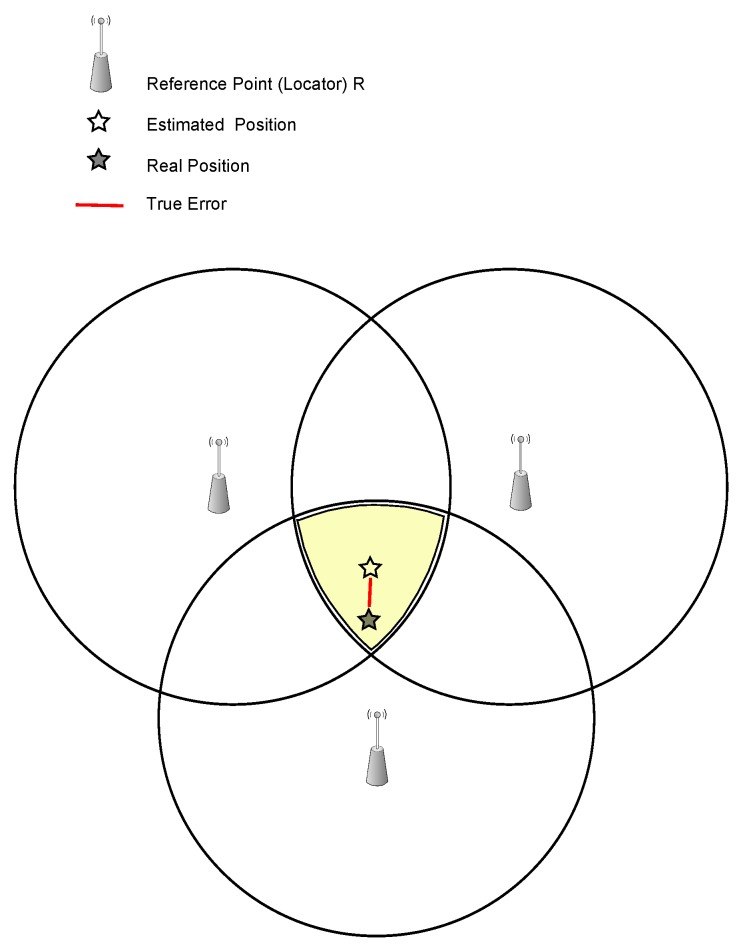
Positioning using reference points.

Indoor positioning systems (IPSs) determine the position of an object in a physical space continuously and in real-time, see Figure 1. IPSs use numerous positioning approaches, which vary greatly in terms of accuracy, cost, precision, technology, scalability, robustness and security [2,5]. Due to the increased demand for accurate indoor positioning, it has become an active research area in which different solutions have been proposed [6]. Many of these solutions utilize some existing technologies to address the problem of position determining. Indoor positioning has its own requirements that differentiate it from outdoor positioning. There are five main quality metrics of indoor positioning systems: (1) system accuracy and precision; (2) coverage and its resolution; (3) latency in making location updates; (4) building’s infrastructure impact; and (5) effect of random errors on the system such as errors caused by signal interference and reflection [7].

Indoor positioning has many applications such as providing indoor navigation systems for blind and visually impaired people, locating devices through buildings, aiding tourists in museums, finding an emergency exit in a smoky environment, tracking kids in crowded places, and tracking expensive equipment. Indoor positioning applications may require different quality attributes, and thus IPSs should be carefully selected to meet the requirements of the application. There are two main questions that need to be addressed by the developers of indoor positioning systems: (1) what are the suitable technologies for implementing the desired IPS? and (2) how can we achieve the most appealing trade-off between the different quality metrics in order to obtain an effective IPS?

Indoor location-based services are an important application of indoor ubiquitous computing. Accurate position measurement is a critical requirement for indoor positioning techniques. Given that ultra wideband (UWB) is a key technique that has proven effective in indoor positioning, a comparative analysis of the state-of-the-art UWB indoor positioning systems is indeed necessary. Furthermore, due to the U.S. Federal Communications Commission’s (FCC’s) recent allowance for the use of unlicensed UWB communications, UWB civilian applications have been studied and explored intensively worldwide. Also, the development of international wireless communication standards that adopt UWB technology has encouraged research and development efforts on UWB. Consequently, developing new algorithms to improve UWB positioning performance is emerging as an active research area [8].

This work is motivated by the fact that UWB is the most promising technology for indoor positioning and tracking. Postioning using UWB technology is still an active and open research area. In order to check how much this area of research is active, we query three main academic databases and search engines; Thomson Reuters, Google Scholar and ProQuest. We limited the search for the last five years (2010–2014) in which the title should include the word “UWB” and either “Localization” or “Positioning”. We only included academic publications (*i.e.*, published at journals and conferences), books, book chapters, patents, and magazine articles. The results of our search are shown in Table 1. Further, to the best of our knowledge, this work is the first analytical study of the state-of-the-art UWB indoor positioning systems. Our study analyzes a wide range of positioning algorithms that have empowered UWB positioning systems through meeting the different applications requirements. The nature of the application in question plays a major role in determining the appropriate solution for achieving certain quality attributes. Hybrid positioning approaches have future potential because they combine features of different mechanisms to improve performance.

**Related Surveys:** A summary of related surveys is given in Table 2. Although several surveys have been conducted to study indoor positioning technologies in general, to the best of our knowledge, there is no survey in the area of UWB positioning algorithms or technologies. Also, no study in the literature compared UWB positioning with other competitive technologies. We believe a survey of emerging UWB indoor positioning technologies will help understand the numerous recent developments in this area.

Contributions. This paper is an extended version of our papers published in [8,19]. Our contributions include the following:

- We provide an updated survey and a comparative analysis of existing indoor positioning technologies that we believe would spur further exploration by the research community of this difficult problem space (see Section 2).
- We provide an updated literature review for UWB positioning systems in particular (see Section 3 and Section 4).
- We conduct a strengths, weaknesses, opportunities and threats (SWOT) analysis for UWB technology, which provides new directions and deeper insights into the state of this technology beyond its well-known pros and cons (see Section 5).

## 2. Indoor Positioning Systems

An indoor positioning system (IPS) is a system that continuously and in real-time determines the position of a person or an object in an indoor environment [5] and has various applications [6,15,20]. IPSs can be used for different private home applications including detecting and tracking of items, providing assistance for elderly and disabled people in their daily activities, and facilitating medical monitoring for vital signs and emergencies. Public buildings (e.g., malls and museums) can be targeted for various useful applications of IPSs such as providing indoor navigation systems for blind and visually impaired people, aiding tourists in museums, and tracking kids in crowded places. Medical care in hospitals is also an important application area for IPSs, as they can be used for tracking patients, tracking expensive equipment to prevent thefts, and conducting precise positioning for robotic assistance during surgeries. Furthermore, IPSs can be used by police and fire-fighters for rescue operations. Tracking fire-fighters in a building on fire is crucial to managing the operation and taking immediate action to rescue them if needed. Also, IPSs can be used for detecting the location of trained police dogs to find explosives inside buildings, to locate stolen products, or to find an emergency exit in a smoky environment. With the development of automation and control, some industries are relying more on IPSs for their operations such as industrial robots, robotic guidance, smart factories, and robot cooperation.

### 2.1. Why Indoor Positioning Systems?

**Figure 2:**
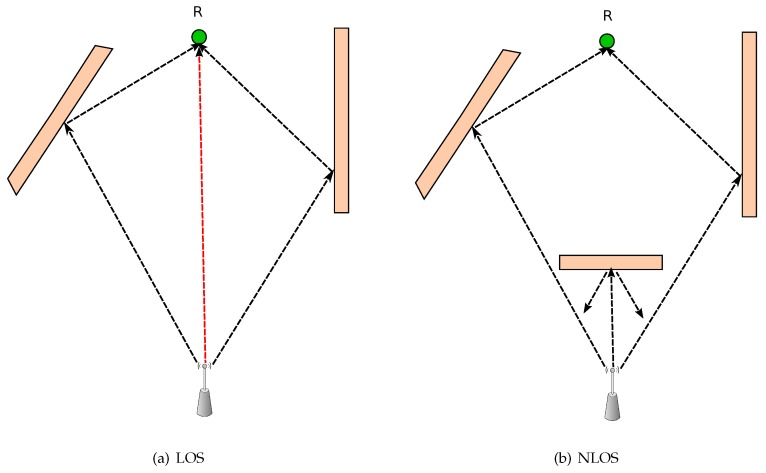
(**a**) Line-of-sight (LOS) *vs*. (**b**) non-line-of-sight (NLOS).

There are many characteristics that make indoor positioning different from outdoor positioning [20]. In comparison with outdoor environments, indoor environments seem more complex because there are multiple objects (such as pieces of equipment, walls, and people) that reflect signals and lead to multi-path and delay problems. Also, due to the existence of various objects, indoor environments typically rely on non-line-of-sight (NLoS) propagation in which signals cannot travel directly in straight path from an emitter to a receiver which causes inconsistent time delays at the receiver, see Figure 2. Furthermore, the existence of objects leads to high attenuation and signal scattering. Indoor positioning suffers from signal stability, as signal strength tends to fluctuate easily due to the existence of many interference sources around us such as mobile devices, Bluetooth devices, Zigbee devices, WiMAX devices, wireless devices, cordless phones, microwave ovens, and fluorescent lights [21].

Relative to outdoor environments, indoor environments are subject to structural movements in which structures including reference points may simply be moved from one place to another. This could tune and calibrate the positioning system to cope with recent changes in the structure. Typically, indoor positioning applications require higher precision and accuracy than outdoor positioning applications to deal with relatively small areas and existing obstacles.

On the other hand, there are some characteristics of indoor environments that facilitate positioning [20]. For example, several factors can help in facilitating positioning within a small coverage area: predetermined infrastructure, corridors, entries and exits, small temperature and humidity gradients, and slow air circulation. Also, indoor environments are less dynamic because objects move at a slower speed within them.

### 2.2. IPS Performance Metrics

IPSs use numerous positioning mechanisms that vary tremendously in terms of cost, accuracy, precision, technology, scalability, robustness, and security [2,3,20]. Some applications may require low-cost IPS, whereas others may require high accuracy IPS such as medical tracking, industrial environmental tracking, and indoor navigation systems for blinds. In this section, we describe different performance metrics of IPSs (also see Table 3).

**Accuracy:** The term accuracy has been defined by the Joint Committee for Guides in Metrology (JCGM) as *“the closeness of agreement between a measured quantity value and a true quantity value of a measure”* [20]. Therefore, the accuracy of an IPS is the average Euclidean distance between the estimated position and the true position [3]. Accuracy is still a very challenging area for many researchers [15]. While the key driver for the majority of applications is IPS accuracy, some compromises might need to made between accuracy and other performance metrics [3,20].

**Availability:** This is the time percentage through which the positioning service is available, taking into consideration the needed accuracy and integrity. An IPS Integrity is the confidence of the IPS output. The availability might be affected by some factors such as communications congestion and routine maintenance. Generally, availability can be seen as three levels; low availability (if <95%), regular availability (if between 95% & 99%), and high availability (if >99%) [15].

**Coverage Area:** This is the area that is covered by the IPS. Each IPS has a particular range. Those that cover wider ranges are considered effective systems [15]. Generally, for positioning systems, there are three levels of coverage: local, scalable, and global [20]. Local coverage refers to a limited area that is well-defined and is not extendable such as a building, while scalable coverage refers to the ability of a system to increase the area by adding hardware. On the other hand, global coverage refers to a system that has a worldwide area such as GPS. Nowadays, existing IPSs ranges are usually from 5 to 50 m. Therefore, building systems with >60 m coverage is difficult [15].

**Scalability:** Although the positioning system can locate objects in various ways (e.g., throughout a campus, in buildings, within a metropolitan area), the number of objects the system might be able to position over a given time is limited [1]. Scalability of an IPS means the system ensures the normal positioning function when it scales in one of two dimensions: geography and number of users. The scale of the number users indicates that the number of units located per time period per geographic area increases [3].

**Cost:** The cost of an IPS can be measured by different dimensions: money, time, space, and energy. These can be affected at various levels of the system: system installation and maintenance, infrastructure components, and positioning devices [3,5]. The cost for system installation and maintenance includes cost required for installation, and any expenses that are required to maintain the system functionality, whereas the cost for infrastructure components and positioning devices may include the costs of buying components and preparing them, as well as the space and energy needed to run those components. Some IPSs, particularly those that reuse existing infrastructures such as the network, are more cost-effective. Some positioning devices, such as passive RFID tags, are completely energy passive, while others consume more energy. Energy is considered a critical resource in IPSs to avoid service disruption and provide higher mobility solutions.

**Privacy:** Privacy is important to individuals using IPSs because a strong access control over how users’ personal information is collected and used is crucial [5]. In order to improve users’ privacy, security mechanisms should be implemented and maintained to protect data from intrusion, theft, and misuse. Unfortunately, the privacy aspect of IPSs was not addressed sufficiently in the indoor positioning literature [15].

### 2.3. Indoor Positioning Technologies

Various indoor positioning technologies can be used concurrently to gain the advantages of each one. The appropriate indoor positioning technology should be selected carefully in order to make the right balance between the complexity and the performance of IPSs [2,5]. Indoor positioning technologies are classified by researchers in many different ways. In 2003, Collin *et al.*, classified indoor positioning technologies into two classes according to the need for hardware: technologies that require special hardware in the building and self-contained technologies [22]. On the other hand, Gu *et al.*, provided different classifications of indoor positioning technologies in 2009, in which they divided them, into two classes based on their need for existence of networks: network-based and non-network-based technologies [5]. The authors also classified indoor positioning technologies according to system architecture into three classes: (1) self-positioning architecture, in which objects calculate their positions by themselves; (2) infrastructure positioning architecture which estimates the locations of the targets using the infrastructure to find if the target is in the coverage areas and track it; and (3) self-oriented infrastructure-assisted architecture which depends on the system that calculates positions and then sends them to the tracked target in response to its request. In addition, they classified indoor positioning technologies into six classes based on the main medium for determining positions: (1) infrared (IR) technologies; (2) ultra-sound technologies; (3) radio frequency (RF) technologies; (4) magnetic technologies; (5) vision-based technologies; and (6) audible sound technologies.

In 2011, Al Nuaimi and Kamel classified indoor positioning technologies into fixed indoor positioning systems and indoor pedestrian positioning systems [15]. This classification is quite similar to the classification introduced by Collin *et al.*, Similarly, Chliz *et al.*, classified the indoor positioning techniques into two categories; parametric where a position is computed based on prior knowledge and non-parametric where a position is computed by processing the data taking into consideration some statistical parameters [23].

On the other hand, Rainer Mautz provided a different classification of indoor positioning technologies in 2012 [20]. He divided them into thirteen categories; camera, infrared, tactile polar systems, sound, WLAN and WiFi, RFID, ultra wideband, high sensitivity GNSS, pseudolites, other radio frequencies, inertial navigation, magnetic systems, and infrastructure systems. Table 4 summarizes existing classification of indoor positioning technologies gathered from the literature.

**Figure 3:**
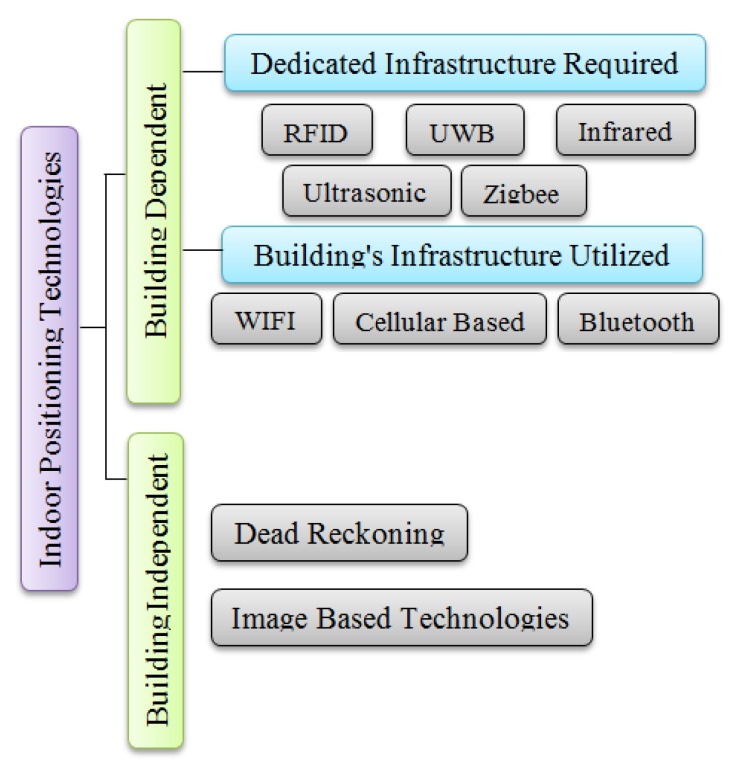
Classification of indoor positioning technologies.

In contrast to the previous classifications, we provide a new classification for indoor positioning technologies according to the infrastructure of the system that uses them, see Figure 3. We classify indoor positioning technologies into two main classes; building dependent and building independent. Building dependent indoor positioning technologies refer to technologies that depend on the building that they will operate in. They depend either on an existing technology in the building or on the map and structure of the building. Building dependent indoor positioning technologies can be further divided into two major classes: indoor positioning technologies that require dedicated infrastructure and indoor positioning technologies that utilize the building’s infrastructure. The need for dedicated infrastructure is determined according to the general structure of most current buildings; e.g., most buildings contain WIFI while almost none contains radio frequency identification. Indoor positioning technologies that require dedicated infrastructure are (1) radio frequency that is either RFID or UWB; (2) infrared; (3) ultrasonic; (4) Zigbee; and (5) laser. Indoor positioning technologies that utilize the building’s infrastructure are (1) WIFI; (2) cellular based; and (3) Bluetooth. On the other hand, the building independent technologies do not require any special hardware in a building such as dead reckoning and image based technologies. In dead reckoning, an object can determine its current position by knowing its past position, its speed and the direction in which it is moving [24]. Image based technologies mainly rely on a camera (e.g., sensor and image processing). Image based technologies can be building independent or building dependent. Image based building dependent technologies depend on special signs in a building or a map of the building. Image based building independent technologies do not require information about the building’s map or any special signs. Figure 3 shows our classification of indoor positioning technologies according to the infrastructure of the system that uses them. Further detail of each technology is given in the following section.

**(1) Radio Frequency Identification (RFID).** Radio frequency Identification uses radio waves to transmit the identity of an object (or person) wirelessly. RFID technology is most commonly used to automatically identify objects in large systems. It is based on exchanging different frequencies of radio signals between two main components: readers and tags. Tags emit radio signals that are received by readers and vice versa. Both tags and readers use predefined radio frequencies and protocols to send and receive data between them. Tags are attached to all the objects that need to be tracked. The tags consist of a microchip which can typically store up to 2 kilobytes of data, and a radio antenna. There are two types of tags; active tags and passive tags. On the other hand, an RFID reader consists of different components; including an antenna, transceiver, power supply, processor, and interface, in order to connect to a server [3,25]. Although different positioning methods can be used with RFID, proximity is the most used one and it senses the presence of RFID tags rather than the exact position [3,20,26]. Also received signal strength (RSS) could be used with RFID [20].

**(2) Ultra Wideband (UWB).** The Federal Communications Commission defines UWB as an RF signal occupying a portion of the frequency spectrum that is greater than 20% of the center carrier frequency, or has a bandwidth greater than 500 MHz. UWB is a communication channel that spreads information out over a wide portion of the frequency spectrum. This allows UWB transmitters to transmit large amounts of data while consuming little transmit energy [25]. UWB can be used for positioning by utilizing the time difference of arrival (TDOA) of the RF signals to obtain the distance between the reference point and the target [27].

**(3) Infrared (IR).** Infrared wireless communication makes use of the invisible spectrum of light just below the red edge of the visible spectrum, which makes this technology less intrusive than indoor positioning that is based on visible light [20,25]. IR can be used in two different ways; direct IR and diffuse IR. Infrared Data Association (IrDA) is an example of direct IR that uses a point-to-point ad-hoc data transmission standard designed for very low-power communications. IrDA requires line of sight communication between devices over a very short distance and up to 16 Mbps. On the other hand, diffuse IR has stronger signals than direct IR, and therefore it has a longer reach (9–12 m). Diffuse IR uses wide angle LEDs which emit signals in many directions. Thus, it allows one to many connections and does not require direct line of sight [25]. Proximity, differential phase-shift, and angle of arrival (AoA) positioning methods are frequently used with Infrared technology [28,29,30].

**(4) Ultrasonic.** An ultrasound wave is *“a mechanical wave that is an oscillation of pressure transmitted through a medium”* [25]. It does not interfere with electromagnetic waves and has relatively short range. Ultrasonic positioning systems leverage building material and the air as a propagation medium [20]. The relative distance between the different devices can be estimated using time of arrival (ToA) measurements of ultrasound pulses traveling from emitters to the receivers. The emitter’s coordinates can be estimated by multilateration from three (or more) ranges to some fixed receivers (deployed at known locations) [20].

**(5) Zigbee.** The ZigBee standard “*provides network, security, and application support services operating on top of the IEEE 802.15.4 specification*” [25]. It is a short distance and low rate wireless personal area network [20,27]. A basic ZigBee node is small and has low complexity and cost. It consists of a microcontroller and a multichannel two-way radio on one piece of silicon [25]. Zigbee is designed for applications that require low power consumption and low data throughput [20]. There are two different physical device types used for ZigBee nodes; full function device (FFD) and reduced function device (RFD) [25]. This technology achieves positioning by coordination and communications with neighbouring nodes. Usually, RSS values are used to estimate a distance between Zigbee nodes [20]. Phase shift measurement is a new approach that was recently introduced to ranging the nodes in ZigBee network [31,32]. The phase shift of the reflected signal from the target node due to the time delay between the target and transmitter is used to measure the distance between them.

**(6) Wireless Local Area Network (WLAN).** The IEEE 802.11 WLAN standard was ratified in June 1997. The standard defines *“the protocol and compatible interconnection of data communication equipment via the air in a local area network (LAN) using the carrier sense multiple access protocol with collision avoidance (CSMA/CA) medium sharing mechanism”* [25]. Using a typical gross bit rate of 11, 54, or 108 Mbps and a range of 50 to 100 m, IEEE 802.11 is considered the dominant local wireless networking standard [3]. Using WiFi in indoor positioning and navigation systems depends on knowing a list of wireless routers that are available in an area in which the system operates. The most popular WLAN positioning method is received signal strength (RSS) which is easy to extract in 802.11 networks and could run on off-the-shelf WLAN hardware [20]. Time of arrival (ToA), time difference of arrival (TDoA), and angle of arrival (AoA) mechanisms are less common in WLAN because of the angular measurements and time delay complexity. Using RSS, the accuracy of WLAN positioning systems is around 3 to 30 m [3].

**(7) Cellular Based.** Global System for Mobile Communications (GSM) networks are available in most countries and can outreach the coverage of WLAN with lower positioning accuracy. GSM operates in the licensed bands and prevents interference from other devices operating at a similar frequency (unlike WLAN) [20]. It is possible to use indoor positioning on a mobile cellular network if the building is covered by one or more base stations with strong RSS [3]. The most common method of GSM indoor positioning is fingerprinting which is based on the power level (RSS) [20].

**(8) Bluetooth.** Bluetooth is a proprietary format managed by the Bluetooth Special Interest Group (SIG) and it represents a standard for wireless personal area networks (WPANs) [20]. Bluetooth is designed to be a very low power technology for peer-to-peer communications, and it operates in the 2.4-GHz ISM band. In comparison with WLAN, the gross bit rate is lower and the range is shorter (approximately 10 cm to 10 m [3,25]). The Bluetooth SIG groups include a local group that investigates the use of Bluetooth wireless technology for positioning [25]. Bluetooth technology commonly uses proximity and RSS methods to estimate positions [25].

**(9) Dead Reckoning.** In dead reckoning, an object can approximately determine its current position by knowing the past position and the velocity with which it moves. Dead reckoning is a navigation technology that needs to begin with a known position; and will then add and track changes. These changes can be in the form of *Cartesian* coordinates or velocity. With the right number of absolute position updates, dead reckoning’s linearly growing position errors might be contained within pre-defined bounds [24]. In order to improve accuracy and reduce error, dead reckoning must use other methods to adjust the position of the object after each interval [33]. Pedestrian dead reckoning is an example that simply estimates the step length and direction of a walking person [24].

**(10) Image Based Technologies.** Image based indoor positioning technologies, which are sometimes called optical methods, include camera and computer vision based technologies [20,34]. Different types of camera can be used such as mobile phone cameras, omni-directional camera, and three dimensional cameras; however, their performance varies due to the amount of information that can be extracted from their images [27]. The success of image based technologies relies on different factors, such as; improvement and miniaturization of actuators, advancement in the technology of the detectors, an increase in the data transmission rates and computational capabilities and development of algorithms in image processing [34]. Image based positioning systems can be categorized into two main categories; *egomotion* systems which use a camera’s motion relative to a rigid scene to estimate the current position of the camera and *static sensor* systems which locate moving objects in the images.

**(11) Pseudolites.** Since Satellites signals cannot penetrate most indoor environment such as buildings, coal mines, long tunnels and others, pseudolites are used to generate GPS-like signals that can be used within indoor environments to allow GPS device to continue receiving signals from those transmitters rather than satellites. In order to cope with less accurate clock within pseudolite transmitters which yields clock bias error, different techniques were developed. Pseudolite-based indoor navigation may differ from system to another depending on the transmitting devices such as pseudolites, synchrolites, locatalites, and transceivers [35]. Wang have presented a survey of historical pseudolite developments including pseudolite-base positioning and technical challenges [36]. Similarly, Eriksson and Badea studied different pseudolite-based indoor navigation systems and provided some recommendations [35]. Pseudolites for indoor environments are still negatively affected by multipath, signal interference among pseudolites, weak time synchronization due to less accurate clocks within pseudolites, and carrier phase ambiguities [35]. Several pseudolites based positioning systems were developed recently that vary in their accuracy and coverage [37,38,39,40].

Indoor positioning applications may require different quality attributes (performance metrics). Therefore, IPSs should be carefully chosen to meet the requirements of the application. Table 5 provides a comparison between indoor positioning technologies in terms of advantages and disadvantages of each technology that needs to be considered during the IPSs selection process.

## 3. UWB Positioning

UWB is one of the most recent, accurate, and promising technologies [44]. The precursor technology of UWB is referred to as a base-band, impulse, and carrier-free technology. The US Department of Defense was the first to use the term ultra wideband. UWB became commercially available in the late 1990 [44]. UWB radio is a method of spectrum access that can provide high speed data rate communication over the personal area network space. UWB is based on transmitting extremely short pulses and uses techniques that cause a spreading of the radio energy (over a wide frequency band) with a very low power spectral density [44]. This high bandwidth offers high data throughput for communication. The low frequency of UWB pulses enables the signal to effectively pass through obstacles such as walls and objects.

There are three main application areas for using UWB: (1) communication and sensors; (2) positioning and tracking; and (3) radar [44,45]. UWB positioning techniques can in fact give real-time indoor precision tracking for several applications such as mobile inventory and locator beacons for emergency services, indoor navigation for blind and visually impaired people, tracking of people or instruments, and military reconnaissance. UWB signals provide accurate position and location estimation for indoor environments [44,46].

### 3.1. Why UWB Has Gained Attention Recently?

In general, UWB has different features that are explored in the literature [3,44,47]. The high data rate of UWB can reach 100 Megabits per second (Mbps), which makes it a good solution for near-field data transmission. Also, the high bandwidth and extremely short pulses waveforms help in reducing the effect of multipath interference and facilitate determination of TOA for burst transmission between the transmitter and corresponding receiver, which makes UWB a more desirable solution for indoor positioning than other technologies [25,46,48]. The duration of a single pulse determines the minimum differential path delay while the period pulse signals determines the maximum observable multipath delay in order to unambiguously perform multipath resolution. In addition, the low frequency of UWB pulses enables the signal to effectively pass through obstacles such as walls and objects which improves accuracy. In fact, UWB provides a high accuracy rate that can minimize error to sub-centimeters. Therefore, UWB is considered to be one of the most suitable choices for critical positioning applications that require highly accurate results.

UWB technology, unlike other positioning technologies such as infra-red and ultrasound sensor, does not require a line-of-sight and is not affected by the existence of other communication devices or external noise due to its high bandwidth and signal modulation [49,50]. Furthermore, the cost of UWB equipment is low and it consumes less power than other competitive solutions.

Many IPSs were implemented commercially using UWB. One well-known positioning system that uses UWB is the *Ubisense* system. In a Ubisense system, a user carries tags that transmit UWB signals to fixed sensors that use the signals to determine the user’s positions using time of arrival (TOA) method [51].

According to a report published by TechNavio market research company, the market of indoor positioning services is expected to grow at a compound annual growth rate of 29.7% over the period from 2014 to 2019 and will be used for various applications in hospitals, shopping malls, airports, museums, athlete training and others [52]. Due to the increase in demand, companies start to explore new opportunities of this new market to leverage the advantages of UWB technology in providing more innovative solutions.

One of the UWB military applications is Alereon that has been used for defense contractors and government agencies to enable wireless integration of positioning equipment and objects. Alereon’s UWB positioning system provides information about devices, weapon and smartphones, and facilitate soldier detection [53].

Ubisense presented a new innovative UWB-based solution to help manufacturers to maintain continuous flow, reduce error and improve efficiency in assembly processes by collecting location and systems data which provides real-time operational awareness. The solution has been successfully adopted by BMW in its facility at Regensburg, Germany [54].

Decawave is another company that uses UWB technology and TOA algorithms to determine the distance among devices and fixed-location beacons to help in different applications such as inventory management, production flow monitoring and management, retail sales monitor and customer behaviour [55]. Integrating UWB chips inside smartphones are demonstrated by a new start-up company called BeSpoon without causing any interference to the way the smartphones work. This integration open a big range of useful applications such as finding someone’s belonging, avoiding leaving smartphones behind, and customizing smartphones based on current indoor location [56].

### 3.2. Signal Modulation

Signal modulation is the process of carrying information on the impulse signal (the carrier signal) by modifying one or more of the signal properties. In general, signal modulation can be categorized based on the signal state into three categories; binary modulation, ternary modulation, and *M-ary* modulation. Signal modulation can also be categorized based on signal properties that need to be modified into four categories; amplitude modulation, frequency modulation, phase modulation, and hybrid modulation.

Signal modulation is a crucial phase in signal transmission that can greatly improve the quality of transmitting signals to achieve certain quality criteria. For example, UWB signals are usually transmitted in the existence of other signals in the air as well as reflected signals that may cause multi-path interference. Thus, UWB must have high modulation efficiency, as signals must be recognized correctly in the presence of noise and interference [45].

Various signal modulations that are used for UWB, such as pulse position modulation (PPM), on-off Keying (OOK), pulse amplitude modulation (PAM), and pulse width modulation (PWM) [45,57]. Signal modulation is utilized to enhance the accuracy of UWB localization [45]. Time-hopping spread spectrum (TH-SS) impulse radio in UWB can be used to solve multipath problems and generate UWB signals with relatively low computational cost. Other modulations can also be used by UWB, such as pseudo random (PR) time modulation, binary phase shift keying (BPSK), time-hopping binary phase shift keying (TH-BPSK), time-hopping pulse position modulation (TH-PPM), and minimum-shift keying (MSK) [7,58]. Further details about using these modulation technologies in positioning are presented in the following sections.

### 3.3. Policy and Regulation of UWB Use

UWB applications must limit their operation to short ranges of frequencies with wide frequency range of UWB to reduce the probability of having interference. In order to regulate the use of the wide range of UWB frequency, license-exempt (unlicensed) and individually licensed frameworks were developed. Several countries and administrations have adopted license-exempt frameworks for UWB communication such as United States, European Union, and many Asia-Pacific countries which are summarized in Table 6. These frameworks require application of special spectral masks and operational conditions. The Federal Communications Commission, European countries, Korea, and Japan are aligned in having the entirely or parts of the 3100 to 10,600 MHz band for such pervasive applications.

In the United States, there are very strict requirements for the bandwidth and power spectral density of UWB systems. The prescribed transmit frequencies are regulated by the National Telecommunications and Information Administration (NTIA) [59]. One of challenges of UWB system implementation is avoiding transmission of the signals at the proscribed frequencies according to the country’s regulation regarding the frequency in which it will be used. Many countries do not provide UWB frequency allocation for a new device unless it achieves the NTIA guidelines on spectrum complaints or any equivalent requirements in other developed countries [60].

## 4. UWB Positioning Algorithms

UWB technology is well suited for indoor positioning applications. In order to employ this technology, different positioning algorithms have been developed in which position information is extracted from radio signals traveling between the reference nodes and target node in addition to the position information of the reference nodes. There are many positioning algorithms that can be classified into five main categories based on some estimating measurements: (1) time of arrival (TOA); (2) angle of arrival (AOA); (3) received signal strength (RSS); (4) time difference of arrival (TDOA); and (5) hybrid algorithm. We give a detailed review of these algorithms for UWB indoor positioning. Then, we compare the algorithms according to various factors including accuracy, environment, estimation technique, range, purpose of use. A summary and comparison of UWB positioning algorithms is presented in Table 7.

### 4.1. AOA-Based Algorithms

**Figure 4:**
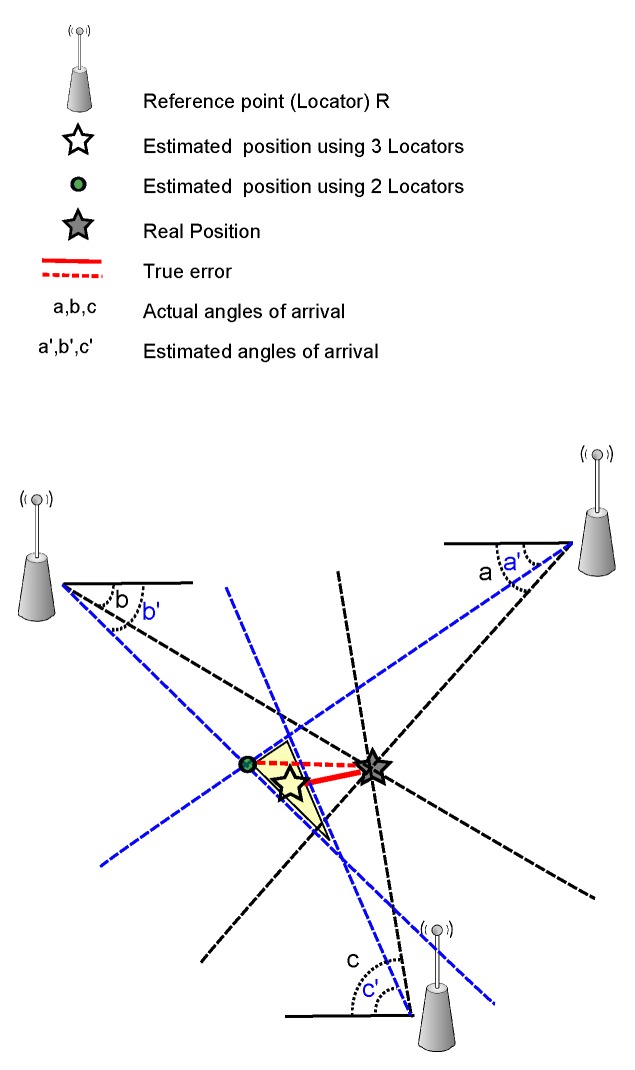
Angle of arrival (AOA)-based algorithms.

In the AOA technique, the estimation of the signal reception angles, from at least two sources, is compared with either the signal amplitude or carrier phase across multiple antennas. The location can be found from the intersection of the angle line for each signal source, see Figure 4. AOA estimation algorithms are very sensitive to many factors, which may cause errors in their estimation of target position. Furthermore, AOA estimation algorithms have a higher complexity compared to other methods. For instance, the antenna array geometry has a major role in the estimation algorithm [93]. Increasing the distance between the sender and receiver may decrease the accuracy [94]. The AOA technique can be used with other techniques to increase its accuracy [95].

AOA based algorithms have been used in a vast amount of literature. Xu *et al.*, presented a new cooperative positioning method based on AOA that utilizes pairwise AOA information among all the sensor nodes rather than relying only on anchor nodes [96]. Lee proposed the use of a signal model and weighted-average to estimate AOA parameters for low data rate UWB (LR-UWB) [97]. A Kalman filter based AOA estimation algorithm was introduced by Subramanian, that relies on a new linear quadratic frequency domain invariant beamforming strategy [98].

Furthermore, many studies have been conducted to evaluate the performance of AOA for different applications, environments, hardware, and configurations. Mok *et al.*, studied the feasibility and performance of AOA for UWB in the Ubisense Real-Time Location System (RTLS) when integrated with GPS to facilitate resource management in underground railway construction sites [99]. The influence of UWB directional antennas on the performance of AOA estimation was analyzed in detail by Gerok *et al.* [100] who presented a corrected AOA estimation algorithm that mitigates the error resulting from the UWB directional antenna.

### 4.2. TOA-Based Algorithms

**Figure 5:**
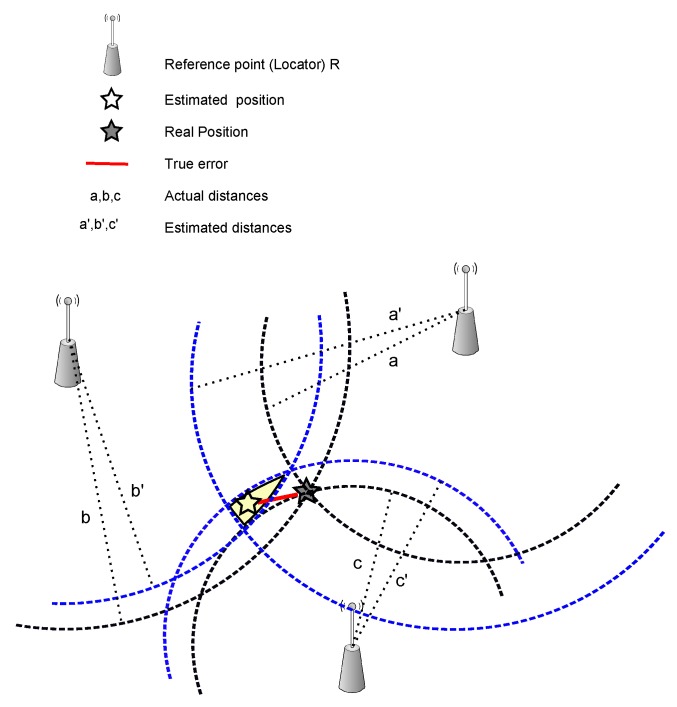
Time of arrival (ToA)-based algorithms.

TOA is based on the intersection of circles for multiple transmitters, see Figure 5. The radius of those circles is the distance between the transmitter and receiver. This distance is obtained by the calculation of the one-way propagation time between them [94]. The time synchronization of all transmitters is required whereas the receiver synchronization is unnecessary; any possibility of significant delay must be accounted for during calculation of the correct distance.

An overview of different positioning techniques for UWB and sources of error for TOA ranging was presented by Dardari *et al.*, who also presented fundamental TOA bounds for ideal and multipath environment [101]. Choliz *et al.*, identified a realistic indoor scenario that is defined by both the layout of walls and corridors, and a specific indoor UWB ranging model to evaluate different kinds of TOA based algorithms for UWB such as weighted least square with multidimensional scaling (WLS-MDS), trilateration, least square with distance contraction (LS-DC), particle filter (PF), and extended kalman filter (EKF) [23].

TOA-based algorithms have been used to locate targeted objects for various applications and environments. Cheng *et al.*, designed a TOA-based personnel localization system for coal mine using UWB technology, which can be very helpful to locate workers effectively in case of accident [46]. For mobile robot tracking, Segura *et al.*, proposed a novel UWB navigation system for the indoor environment that employs a TOA based estimation algorithm to accurately locate the mobile robot [48]. Fischer *et al.*, designed a monolithic integrated transceiver chipset for UWB to use in indoor localization systems where TOA techniques have been used for position estimation [61]. The system was implemented for line-of-sight environments, and its accuracy was estimated to be 8.3 cm. On the other hand, Tom’e *et al.*, designed and built a large deployable UWB-based local positioning system (LPS) in which TOA is used for position estimation [72]. Other systems have utilized other means such as direct current artificially generated magnetic fields in order to determine the location of mobile devices within a given indoor environment [102].

Kok *et al.*, designed an indoor positioning approach in 2015 based on a sensor fusion method that combines inertial sensors and time of arrival measurements from UWB. Their approach depends on an UWB transmitter that is rigidly attached to inertial measurements unit and a number of UWB receivers placed indoors. UWB measurements here are modeled using a heavy-tailed asymmetric distribution that handles the delays of measurements due to NLOS and multipath. In order to obtain information of a position from the UWB measurements, the receivers’ positions must be known and their clocks must be synchronized. Their experiment shows that their UWB measurements model lead to accurate position estimates [82].

Positioning of human body movement for cluttered indoor environment uses wearable UWB technology to obtain 1–2 cm error using eight base stations, while four base stations were used in different shapes to obtain a slightly lower accuracy for locating body movement [84]. In most configurations, peak detection algorithm was used to estimate TOA of the received signals.

In 2013, Zaric *et al.*, presented the ability of localization of a conformal wall-embedded tag in a suitcase using UWB. The system contains two main modules: an optical position measurement system that is based on a web-camera and an UWB positioning system. The author attempted to test the localization accuracy and the tag detection reliability in different situations of a suitcase. The test shows average positioning error of around 8 cm [85].

Generalised Gaussian mixtures (GGM) approximative method was compared and outperformed extended Kalman filter to provide more accurate position estimation in movement tracking in environment with uncertainty while still keeping computational complexity reasonable to use in mobile devices [89].

### 4.3. TDOA-Based Algorithms

**Figure 6:**
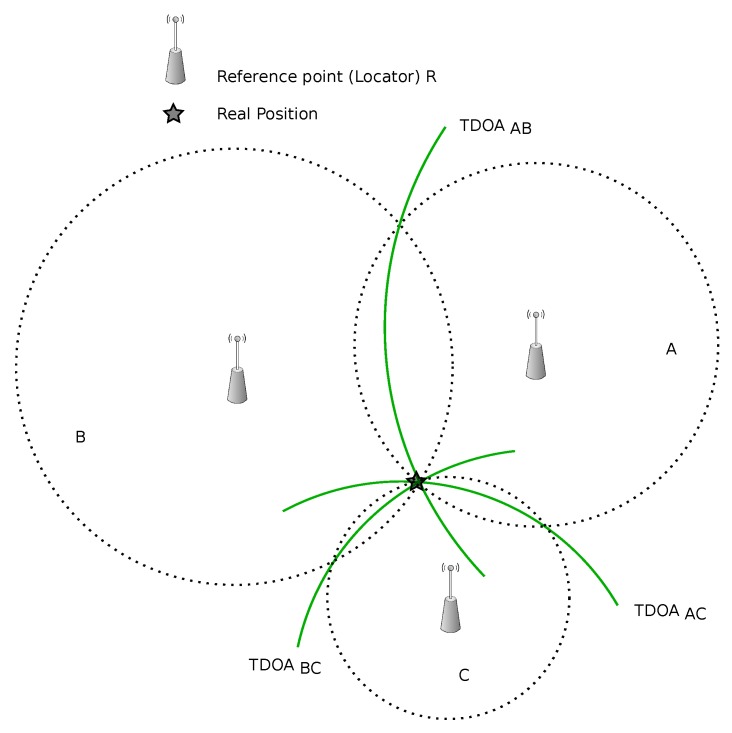
Time difference of arrival (TDoA)-based algorithms.

TDOA is based on measuring the time difference of arrival of a signal sent by an object and received by three or more receivers, see Figure 6. In this manner, the location of the object (transmitter) will be determined. Also, the scenario can be flipped so a single receiver can determine the target location by measuring the delta in arrival times of two transmitted signals [94]. Typically, only one transmitter is available that requires the multiple receivers to share the data and cooperate to determine the location of the transmitter. This cooperation requires significant bandwidth in comparison with other algorithms.

Krishnan *et al.*, have used TDOA for UWB indoor positioning system where the site has been divided into cells and each cell has four UWB readers mounted on the top corners to have line-of-sight with user tag. In this manner, the readers will be able to receive the signals from the user tag then send the time of arrival to a central processing unit to determine TDOA and find user location [50]. Rowe *et al.*, designed one dimensional system with two sensors and one tag using TDOA-based algorithm to determine the tag location [62]. On-off keying (OOK) modulation was used to overcome the collision induced by synchronous tag transmission, increase the performance, and decrease the cost and power at the same time. Leitinger *et al.*, utilized prior knowledge of floor plan to improve positioning in multipath environment using the concept of equivalent Fisher information [103]. Cyganski *et al.*, presented a new way to utilize multi-carrier signal to performance degradation due to multipath signals within indoor environment [104,105]. The authors applied matrix decomposition-based multi-carrier range recovery algorithm to improve accuracy of positioning in severe multipath environment.

In 2012, Ruiqing Ye presented a detailed study about UWB localization systems that have different accuracy requirements and complexity. He developed a three dimensional localization system with a centimeter accuracy using UWB technology to track miniature mechanical parts in an airplane wheel. The system uses a TDOA algorithm and four receivers in order to track these parts. Two technical challenges are observed after testing the system in an environment that is rich with metal objects: angle-dependent waveform distortion and path overlap. He proposed a range estimation method to reduce the error caused by the path overlap. Also, the author discussed the effect of the receiver configuration on the performance of TDOA. Moreover, the author designed a wireless localization system that has a centimeter accuracy [86].

Zwirello *et al.*, provided a complete demonstration of designing an UWB positioning system in 2012 that includes a choice of positioning method, access points’ placement, error sources analysis, and simulation and verification of measurement. The authors also implemented and evaluated various TDOA algorithms. They concluded that a combination of modified Bancroft and Levenberg Marquardt algorithms are the most efficient algorithms. A series of evaluations and tests were conducted in designing the corresponding UWB positioning system. They improved the average accuracy from 9 to 2.5 cm [87].

Garcia *et al.*, presented a robust UWB indoor positioning to operate in a highly complex indoor scenario in which NLOS condition is highly expected [91]. The system detects the NLOS condition using channel impulse response in order to effectively apply Extended Kalman Filter that improves the accuracy.

### 4.4. RSS-Based Algorithms

In RSS-based algorithms, the tracked target measures the signal strength for received signals from multiple transmitters in order to use signal strength as an estimator of the distance between the transmitters and receivers. This way, the receiver will be able to estimate its position relative to the transmitter nodes. Although RSS is sensitive to multipath interference and a small-scale channel effect that causes a random deviation from mean received signal strength, it is used frequently with unrealistic assumptions. For example, the transmitted power and path loss exponent are already known, and the transmitter antennas are isotropic [94,106]. According to Pittet *et al.*, the accuracy of RSS for non-line-of-sight (NLOS) and multipath environment is low, which shows clearly that RSS is not the right estimation method for indoor positioning systems [64]. Gigl *et al.*, explored the performance of RSS algorithms for positioning using UWB technology [107]. They also studied the effect of small scale fading on the system accuracy; however, a simulator based on the UWB channel model 802.15.4a was used to evaluate the algorithms rather than relying on real scenarios for indoor environments. Leitinger *et al.*, used maximum likelihood estimator as well as floor plan information to improve positioning in the existence of diffuse multi path for the NLOS environment [90].

On the other hand, RSS-based algorithms have some advantages over other algorithms which make them attractive in some cases. In such algorithms, the mobile tags act as receivers only and thus rely on the strength of received signals from multiple transmitters to find their positions. In this manner, RSS-based algorithms tend to have less communication traffic which helps in improving channel access control and positioning accuracy. Also, less communication traffic helps to overcome limitation on the number of tags in use. the mobile tags are receivers only. There is no limitation on their numbers.

RSS-based algorithms can be categorized into two main types: trilateration and fingerprinting [108]. Trilateration algorithms use RSS measurements to estimate the distances to three different reference nodes and hence estimate the current location. On the other hand, fingerprinting requires collecting a dataset of RSS fingerprints of a scene, which is later used to match online measurements with the closest fingerprint in the dataset in order to estimate the location.

### 4.5. Hybrid-Based Algorithms

When multiple positioning techniques are used, they can complement each other or target different parts of the site that fit with their strengths. Overall accuracy will increase as well as complexity and cost. Jiang *et al.*, presented a tracking system for staff, patients, and instruments in a hospital environment [63]. They used GPS for outdoor tracking and UWB for indoor tracking. Furthermore, the site was divided into cells, each of which had at least 4 UWB readers and a GPS repeater. They used a PDA that had a built-in GPS receiver and was connected to a UWB tag in order to work with both GPS and UWB at the same time. The UWB subsystem uses both AOA and TDOA received by UWB readers to estimate the user position. Similarly, Kuhn *et al.*, designed a multi-tag access scheme for a UWB localization system, in which minimum-shift keying (MSK) modulation was used with 2.40–2.48 and 5.40–10.6 GHz frequency and a refresh rate of 1–20 Hz in the range of 1–100 m [58]. They also have used time division multiple access (TDMA) for channel access control. TDOA was used to discover new tags and identify their positions in 3D. Experimentally, it uses two tags and switches between them 20 times per second.

Wymeersch *et al.*, presented an overview of cooperative localization approaches for UWB wireless networks [109]. They also presented a new cooperative localization algorithm which map the graphical model of statistical interference onto the current network topology. Although the algorithm is fully distributed, it achieve high localization accuracy with low communication overhead. A new pedestrian navigation solution has been introduced by Pittet *et al.*, that combines a UWB localization system and micro electro mechanical sensors (MEMS) to improve the performance of pedestrian positioning [64]. AOA and TDOA were used to determine the presence of multipath and position estimation. Furthermore, they used an extended Kalman filter (EKF) based algorithms to couple the measurement of these two subsystems to combine the complementary advantages of UWB and MEMS. Another system has been introduced by Shahi *et al.*, that consists of a network of tags and receivers communicating over 68 GHz signals [47]. The path from transmitter to receiver is measured to locate the tag. The true location is determined by the direct path signals; however, the error was produced by reflections of the signals. The direct path signal is distinguished from reflection using UWB, so the accuracy increases. The computation is calculated in one master server that uses AOA and TDOA for estimation. Also, FuCheng and MingJing designed a UWB localization and tracking system based on Kalman, linear H and extended H filters to accurately estimate the target position using DOA and TOA [110]. Their system was implemented in a 30 m × 30 m cell with one access point that is equipped with 4 elements array and noise statistics.

Several other systems have been developed for critical missions to help track people and objects. A UWB indoor/outdoor NLOS localization system has been implemented for disaster aid that uses GPS for outdoor localization and UWB for indoor localization [111]. TDOA and RSS are used to improve localization performance. Another UWB tracking system for athletes has been presented by Mucchi *et al.*, that determines the athlete’s speed and acceleration and analyzes his/her performance after medical surgery [66]. They have implemented their system for outdoor environments with different cell sizes and for indoor environments using 4 sensors. The system was implemented for line-of-sight (LOS) environment setup and uses TOA and AOA for positions’ estimation with good accuracy. DeiBler *et al.*, designed another system that tackles the problem of simultaneous localization and mapping in an emergency like an earthquake, fire, or terrorist attack [69]. The system was designed to perform UWB indoor mapping using a mobile antenna array with two receiver antennas and one transmitter between them. DeiBler *et al.*, used Kalman filter for position estimation and Rao-Blackwellized particle filter for data association and initialization of new objects.

Furthermore, a new UWB indoor navigation system was proposed by Segura *et al.*, that includes two sub-systems: a location system and mobile robot (MR) control system [48]. They detected the first arrival of the signal by designing a novel dynamic threshold crossing algorithm and using TOA/TDOA for estimation. Time division multiple access (TDMA) is used to avoid interference from multiple users.

Several other efforts have been done to improve positioning in UWB using hybrid based algorithms. Digel *et al.*, designed and improved a digitizer of non-coherent impulse radio ultra wideBand (IR-UWB) [112]. Jiang *et al.*, designed a technique to mitigate NLOS error by using biased Kalman filtering (BKF) and maximum likelihood estimation (MLE) where both AOA and RSS were used [71]. Srimathi and Kannan made a comparison between time-hopping spread-spectrum (TH-SS), time-hopping binary phase-shift keying (TH-BPSK), and TH-SS coded and un-coded scheme UWB systems [113]. Zebra is a commercial UWB positioning system that offers a UWB real-time location system (RTLS) integrated with other RTLS, which can use technologies, such as radio frequency identification (RFID), GPS, and wireless local area network (WLAN) [58].

In 2014, Harikrishnan Ravikrishnan discussed the tracking objects on an assembly line problem. The author solved the problem using an ultra wideband positioning system and an particle filter. UWB positioning is noisy; *i.e.*, its accuracy is not sufficient for monitoring objects in an assembly line, therefore the author used an assembly line map to constrain the motion tracking. The assembly line is modeled using a piece-wise function of linear segments. In the experiment, the author compared the functionality of Kalman filter with the functionality of the particle filter. The particle filter gave a 55% improvement in the position estimation while the Kalman filter gave only a 37% improvement [83].

A quality assessment study of Ubisense real-time location system was performed by Perrat *et al.* [92]. The system was used to track wheelchair athletics in an indoor court sports. Wang and Zhang proposed a joint estimation algorithm of TOA and AOA for UWB systems based on sparse representation framework and by using two-antenna receiver. The simulation results show that the new algorithm outperforms traditional methods [88].

### 4.6. Comparison of Positioning Algorithms

After our discussion of the four common positioning algorithms, we present a comprehensive comparison of them, see Table 8. AOA is less practical than the other algorithms due to the difficulty and cost of maintaining the required large dimensions of antenna arrays and sensors. This has been verified by a comparative study of UWB [114,115]. Also, AOA requires strong cooperation between the sensors and is subject to error accumulations [116]. While AOA has an acceptable accuracy, it is less powerful for UWB signals that have strong scattering.

On the other hand, the RSS algorithm does not effectively utilize the high bandwidth of UWB relative to other algorithms. Hatami and Pahlavan showed that RSS is more suitable for systems that use narrowband signals while the TOA algorithm performs better in wideband systems such as UWB-based systems [117]. Using RSS, the large bandwidth has no positive effect in the sense of increasing the achievable accuracy. This makes the RSS method less attractive in comparison to the time-methods that offer high accuracy.

Regarding positioning in a two-dimensional space, the TDOA algorithm requires at least three properly located base stations, whereas the AOA algorithm requires only two base stations for location estimation. In terms of accuracy, small errors in angle measurement will negatively impact accuracy when the target object is far away from the base station. TDOA and AOA location algorithms can be combined in one algorithm in which they complement each other; such an algorithm has advantages achieving high location accuracy [118].

TOA and TDOA have higher accuracy relative to other algorithms because of the high time resolution of the UWB signals [114]. Clock synchronization and clock jitter are important factors that affect the accuracy of the TOA algorithm because clock synchronization is needed between the nodes to estimate the time of arrival accurately. On the other hand, TDOA is a more effective solution if there is no synchronization between the node and the reference nodes when the reference nodes are synchronized among themselves [95,119,120]. Hyper algorithms have been found to be the most effective solutions for UWB positioning systems because they combine the advantages of their algorithms.

## 5. SWOT Analysis

SWOT analysis is a useful analysis tool for understanding and evaluating a technology, solution or business. SWOT analysis aims to identify the key internal (strengths and weaknesses) and external (opportunities and threats) factors that may affect the success of an analyzed target. SWOT analysis has been applied in many areas such as; industry, management and engineering. Here, we apply the SWOT analysis to evaluate UWB in terms of strengths, weaknesses, opportunities and threats to gain a deeper understating of UWB. A summary of the SWOT analysis is shown in Table 9.

### 5.1. Strength

One advantage of using UWB is that it is license-free because of its low power. UWB is not classified as radio equipment because its low power signal does not interfere with most of the existing radio systems [121]. UWB consumes low power in comparison with other positioning technologies that enable power efficiency for better battery life of devices. UWB used pules that allows transmitter to send only during the pulse transmission which in turn produces strict duty cycle on the radio in order to minimize the baseline power consumption [122]. Moreover, the complexity of UWB communication is implemented in the receiver rather than in the transmitter. This feature offers low power consumption for sender and shifts complexity as much as possible to the receiver. In addition, UWB has a very high level of multipath resolution because of its large bandwidth. Large bandwidth provides frequency diversity that makes time modulated ultra wideband (TM-UWB) signal resistant to the multipath problems and interference [121]. Time Modulated UWB has a low probability of interception and detection and it is used in some particular applications such as in the military.

The large bandwidth is the main feature of UWB wireless systems. This feature offers an improved channel capacity and high data rate communication in digital communication systems [123]. The channel capacity is defined by Shannon’s law that the channel capacity is proportional to the bandwidth (B) and the log of signal to noise ratio $\frac{S}{N}$ plus one.

(1) $$C=B\;log_{2}\left(1+\frac{S}{N}\right)$$

In addition to the advantage of large bandwidth, it has potential for high processing gain in communication systems. Processing gain for real direct-sequence of UWB (DS-UWB) modulation systems is defined as two times the ratio of noise bandwidth at the front end of the receiver to the noise bandwidth of the symbol rate. Here is the formula of DS-UWB processing gain [123]:

(2) $$PG=2\times \frac{\mathrm{Noise Bandwidth}}{\mathrm{Symbol Rate}}$$

The large processing gain offers a greater immunity distortion and noise. It allows negative signal to interference and noise ratio (SINR) to be recovered [123].

UWB signals have greater penetration of obstacles (such as walls) than conventional signals, and they achieve the same data rate [124]. However due to power restriction, common UWB positioning systems may face difficulty to penetrate walls. Furthermore, UWB transmissions involve very short pulses, which have recently received significant interest. Very short pulses offer an advantage in terms of resolvability of multipath components [124]. Many received signals in an environment that are characterized by multipath is a superposition of the delayed replicas of the signal. This has been avoided in UWB because the reflections from objects and surfaces near the path between the transmitter and receiver tend not to overlap in time because of the very short pulses of UWB. This means UWB has a desirable direct resolvability of direct multipath components.

UWB technology’s carrierless transmission property offers the advantage of simple and small hardware. UWB transceivers can be built with much simpler radio frequency architecture than narrowband systems with fewer components. Also, there is no need for a power amplifier because of their low power consumption [124]. In general, UWB hardware is considered to be simple and the hardware simplicity depends on the application and operational scenario. For example, the transmitter does not need analog to digital (A/D) converter, digital pulse shaping filter, or equalizer to correct carrier phase distortion [124]. Despite the hardware simplicity of the majority of UWB transceivers, most vendors are unable to produce inexpensive transceivers.

### 5.2. Weaknesses

Although UWB has many strengths for different applications, it has some weaknesses. For example, the possibility of interference with nearby systems that operate in the ultra wide spectrum due to misconfiguration [123]. In the United States, the UWB frequency range for communication applications is 3.1 to 10.6 GHz, which operates in the same frequencies as popular communication products such as Worldwide Interoperability for Microwave Access (WiMAX) and digital TV. In some countries, it may also interfere with systems such as third-generation 3G wireless systems [123]. There are some concerns that several UWB devices may cause harmful interference to GPS and aircraft navigation radio equipment [124]. To overcome those concerns, various techniques have been developed to eliminate harmful interference with other sensitive services, such as Detection and Avoidance (DAA) [123].

Interference may also occur from the existing system to the UWB system. The UWB system’s signals may spread over other bandwidths that contain the existing frequency of a narrowband system [124]. This interference can be elevated by using minimum mean-square error (MMSE) multiuser detection schemes to reject strong narrowband interference. Furthermore, simultaneous ranging among many UWB tags may cause some problems to channel access control which may lead to degradation in positioning accuracy [125].

Although using very short pulses in UWB has many advantages, the UWB receiver requires signal acquisition, synchronization and tracking to be done with very high precision in time relative to the pulse rate. These steps are time-consuming [124]. There are some techniques for reducing this time such as using a preamble sequence for rapid acquisition.

### 5.3. Opportunities

UWB is becoming a choice for many systems that require high accuracy, such as in building robot guidance and tracking systems that utilize its advantages. Furthermore, UWB is used for medical applications that require sub-millimeters of accuracy [51]. In addition, UWB is used in radars in order to improve their high performance [126].

For indoor localization systems, there are multipath reflections from objects inside rooms that negatively impact radio signals. However, UWB signals have time resolution, which offers a high-resolution positioning application for solving the multipath problems [123].

As mentioned before, UWB communication signals have short pulses. Those short pulse signals can be utilized in non-communication purposes [124]. For instance, low-power UWB RFID tag transmitters have been used to locate objects with an accuracy proportional to the inverse of the signal bandwidth.

UWB could be beneficial for industry and service providers in many applications such as sensor, positioning, and identification network (SPIN) systems [127]. These systems require a large number of devices (sensors and tags) in industrial warehouses to transmit low-rate data combined with position information. This allows the devices to operate over a long distance (around 100 m) between mobile tags and sensors of UWB.

There are some challenges with using radio frequency (RF) operation for a shipboard environment. Using UWB and network analyzer measurements offers good opportunities for NLOS communication for indoor and onboard ships [124]. It allows signals to propagate well aboard ships and around objects, which provides reasonable accuracy to determine positions. UWB is used in radar in order to improve its high performance [126].

### 5.4. Threats

UWB usually does not have a negative impact neighboring devices because it uses some techniques to avoid interference with other devices [51]. However, UWB is still commercially slightly expensive compared to other technologies (see [45] for further limitations).

While UWB systems are known to be robust against multipath reflection issues, they are not totally immune to multipath effects [127]. For example, when there is an extreme ratio of link distance to antenna height, this may result in signal losses and propagation delay that lasts to tens or even hundreds of nanoseconds.

The design and implementation of antennas for UWB systems can be more challenging than the bandwidth and variable conditions of operation [127]. This may add some limitations to UWB systems in comparison with conventional RF.

## 6. Lessons Learned and Concluding Remarks

Positioning is one of the most important and challenging phases in navigation systems where different technologies have been developed to improve performance. In this paper, we present an analytic study of UWB positioning, in which a detailed and updated overview of UWB indoor positioning techniques was discussed. Furthermore, we performed a SWOT analysis of UWB technology, that focuses mainly on positioning applications and identifies both internal (strengths and weaknesses) and external factors (opportunities and threats) that affects this technology.

As discussed in the SWOT analysis, while UWB provides a high accuracy positioning in addition to many other features (e.g., license free, low power consumption, does not interfere with most of the existing radio systems, high level of multipath resolution, large bandwidth, and high data rate communication), UWB technology may affect GPS and aircraft navigation radio equipment and can also cause interference to the existing systems that operates in the ultra wide spectrum. In comparison to other technologies, UWB systems have emerged as one of the leading technologies for indoor positioning and have been used in many more applications than before.

Several factors can contribute to the enhancement of positioning performance. For example, a priori knowledge on the environment can improve the positioning performance while cooperation among the nodes may enhance the performance if carefully exploited. Hybrid methods seem promising as they are more tolerant to external side effect of interference and reflection.

## Figures and Tables

**Table 1:** Number of research articles in the last five years for three academic databases and search engines.

Academic Database	2010	2011	2012	2013	2014
Thomson Reuters	38	34	44	62	35
Google Scholar	80	63	70	69	55
ProQuest	79	54	62	67	52

**Table 2:** List of related surveys.

Survey	Year	Environment	Technology	Description
Pandey *et al.* [9]	2006	Indoor, Outdoor	General	Localization techniques for wireless networks
Liu *et al.* [3]	2007	Indoor	Wireless	A quantitative comparison of indoor positioning solutions
Khodjaev *et al.* [10]	2009	Indoor	UWB	A comparison of different methods of NLOS identification and error mitigation
Honkavirta *et al.* [11]	2009	Indoor	Wireless	A comparative study of WLAN location fingerprinting methods based on RSS values
Wang *et al.* [12]	2009	Indoor, Outdoor	General	Localization methods of sensor nodes in WSN
Guvenc *et al.* [13]	2009	Indoor	RF	TOA positioning algorithms in NLOS environments
Ruiz-López *et al.* [14]	2010	Indoor	General	An analytic review of different positioning techniques in relation several quality attributes
Al Nuaimi and Kamel [15]	2011	Indoor	General	A short survey of existing indoor positioning technologies
Ijaz *et al.* [16]	2013	Indoor	RF, Ultrasonic	A comparison of the ultrasonic system based on performance, accuracy and limitations
Adalja Disha [17]	2013	Indoor	Wireless	A performance comparisons including (among others) precision complexity, accuracy, scalability, cost, and robustness
Zhu *et al.* [18]	2014	Indoor	General	A review of indoor positioning technologies

**Table 3:** Performance metrics of indoor positioning systems (IPSs).

Metric	Definition
Accuracy	*“The closeness of agreement between a measured quantity value and a true quantity value of a measure”* [20]
Availability	The positioning service availability in terms of time percentage
Coverage Area	The area covered by an IPS
Scalability	The degree to which the system ensures the normal positioning function when it scales in one of two dimensions: geography and number of users
Cost	Can be measured in different dimensions; money, time, space, and energy which can be affected at different levels of the system: system installation and maintenance, infrastructure components, and positioning devices [3,5]
Privacy	Strong access control over how users’ personal information is collected and used [5]

**Table 4:** Different Classifications of Indoor Positioning Technologies.

Author-Year	Classified based on	Categories
Collin *et al.*—2003	Need for hardware	Technologies that require hardware in the building, and self-contained ones
Gu *et al.*—2009	Existence of network	Network-based and non-network-based technologies
	System architecture	Self-positioning architecture, self-oriented infrastructure-assisted architecture, and infrastructure positioning architecture
	Main medium used to determine positions	Ultrasound, radio frequency, magnetic, vision-Based, and audible sound technologies
Al Nuaimi and Kamel—2011	Installed system in a building	Fixed indoor positioning and indoor pedestrian positioning
Chliz *et al.*—2011	Prior knowledge	Parametric and non-parametric technologies
Rainer Mautz—2012	Sensor type	Camera, infrared, tactile & polar systems, sound, WLAN and WiFi, RFID, ultra wideband, high sensitivity GNSS, pseudolites, other radio frequencies , inertial navigation, magnetic systems , and infrastructure systems

**Table 5:** Comparison between Indoor Positioning Technologies.

Technology	Common Measurement Methods	Advantages	Disadvantages
RFID	Proximity, RSS	Penetrate solid, non-metal objects; does not require LOS between RF transmitters and receivers [25].	The antenna affects the RF signal, the positioning coverage is small, the role of proximity lacks communications capabilities, cannot be integrated easily with other systems [27], RF communication is not inherently secure and consumes more power than IR devices [25].
UWB	ToA,TDOA	High accuracy positioning, even in the presence of severe multipath, effectively passes through walls, equipment, and any other obstacles; UWB will not interfere with existing RF systems if properly designed [25].	High cost of UWB equipment [27]; although UWB is less susceptible to interference relative to other technologies, it is still subject to interference caused by metallic materials [3].
Infrared	Proximity, Differential Phase-shift, AoA	Since IR signals cannot penetrate through walls, it is suitable for sensitive communication because it will not be accessible outside a room or a building [25].	Does not penetrate walls, therefore it is typically used in small spaces such as one room; IR communication is blocked by obstacles that block light which includes almost everything solid [25]; requires LOS between sender and receiver when using direct IR; One problem with diffuse infrared systems is their poor performance in locations with direct sunlight or fluorescent lighting because the infrared emissions (of the light sources) may interfere with the signals [25].
Ultrasonic	ToA, TDOA	Does not require LOS; do not interfere with electromagnetic waves [25]	Does not penetrate solid walls; there may be loss of signal because of obstruction; false signals because of reflections; and interference caused by high frequency sounds (e.g., keys jangling) [25].
Zigbee	RSS, Phase Shift Measurement	Its sensors require very little energy [25,27], and Low cost [25].	ZigBee which operates in unlicensed IS bands seems vulnerable to interference caused by a wide range of signal types (using the same frequency). This might disrupt radio communication [20]; it is suitable for networks in which conversation between two devices takes some few milliseconds which allows the transceiver to switch to sleep mode quickly [25].
WLAN	RSS	Use existing communication networks that may cover more than one building; the majority of devices available nowadays are equipped with WLAN connectivity; WLANs exist approximately in the majority of buildings; LOS is not required [20].	A major drawback of WLAN fingerprinting systems is the recalculation of the predefined signal strength map in case of changes in the environment (e.g., open/closed doors and the moving of furniture in offices). [20].
Cellular Based	RSS	No interference with devices that operate at the same frequency; the hardware of customary mobile phones can also be used [20].	Low reliability due to varying signal propagation conditions [20].
Bluetooth	Proximity, RSS	Does not require LOS between communicating devices [25]; a lighter standard and highly ubiquitous; it is also built into most smartphones, personal digital assistants, *etc*. [3].	The greater the number of cells, the smaller the size of each cell and hence better accuracy, but more cells increase the cost; requires some relatively expensive receiving cells; requires a host computer to locate the Bluetooth radio. Because the 2.4 GHz spectrum that Bluetooth is using is unlicensed, new uses for it are to be expected, and as the spectrum becomes more widely used; radio interference is more likely to occur [25].
Dead Reckoning	Tracking	Does not require additional hardware such as sensors	The DR calculates only an approximate position [41].
Image based technologies	Pattern recognition	They are relatively cheap compared with other technologies such as ultrasound and ultra wideband technologies [42].	Requires LOS, coverage is limited [27].
Pseudolites	RSS	They allow to extend the coverage area much farther to several kilometres and provide great flexibility in deployment that can be optimized for a particular application and they are also compatible with existing GPS receivers [43].	They are negatively affected by multipath, signal interference among pseudolites, weak time synchronization due to less accurate clocks within pseudolites and carrier phase ambiguities [35].

**Table 6:** Policies and regulations enforced by different countries.

Country	License	Freq. Mask	Regulations
US	Unlicensed	3.1–10.6 GHz	Indoor only
			Cannot be used in fixed outdoor environments or those linked to a fixed outdoor antenna
Europe	Unlicensed	3.1–10.6 GHz	Indoor only
			Devices must be installed in road and rail vehicles, transmit power control (TPC) of a range 12 dB relative to the maximum allowed radiated power. The maximum mean e.i.r.p. spectral density must be −53.3 dBm/MHz when no TPC is in place
UK	Unlicensed	3.1–10.6 GHz	In harmony with European regulations
			Indoor
			Outdoor but not attached to fixed installation, infrastructure or automotive vehicle (or railway vehicle)
			The equipment must not cause interference to any wireless telegraphy
S. Korea	Unlicensed	3.1–10.2 GHz	Indoor only
			It is similar to bands allocated by FCC and it uses a different emission mask for accommodating its spectrum environment
			for UWB devices operating in the 3100 to 4200 MHz band, it requires use of detect and avoid technology
Japan	Unlicensed	3.4–10.25 GHz	Indoor only
			In the 3400–4200 MHz band must incorporate interference mitigation techniques
			In the 4200–4800 MHz band it can operate without mitigation techniques
Singapore	Unlicensed	3.4–9 GHz	Indoor only
			The PSD limit shall be −41.3 dBm/MHz for devices equipped with interference mitigation techniques. For devices without mitigation techniques, the permissible PSD limit is −70 dBm/MHz

**Table 7:** Comparison of Ultra WideBand (UWB) Systems.

No.	Authors	Year	Accompanied Technology	Algorithm	Environment	More Details
1	Ch’oliz *et al.* [23]	2011		TOA	LOS, NLOS	Compared the performance of impulse radio (IR) UWB indoor tracking systems using different parametric and non-parametric algorithms such as weighted least square with multidimensional scaling (WLS-MDS), trilateration, least square with distance contraction (LS-DC), particle filter (PF), and extended kalman filter (EKF).
2	Guangliang Cheng [46]	2012		TOA	LOS, NLOS	Presented a new UWB-based personnel localization system for coal mines.
3	Fischer *et al.* [61]	2010		TOA	LOS, NLOS	Designed a new monolithic integrated IR-UWB transceiver chipset with a high-precision TOA measurement unit using two-way ranging and 8-PPM modulation.
4	Krishnan *et al.* [50]	2007		TDOA	NLOS	Used multi-cell implementation to cover large spaces, using Chan’s method to provide an accurate estimate of the mobile tag’s position within each cell. A heuristics-based approach was used to improve the accuracy at the boundaries.
5	Rowe *et al.* [62]	2013		TDOA		Presented a new multi-tag millimeter accuracy localization system that utilize digital sampling to enhance its accuracy.
6	Jiang *et al.* [63]	2010	GPS	AOA, TDOA	LOS, NLOS	Provided indoor/outdoor location tracking in a hospital environment by integrating UWB and GPS technologies in one system. Ubisense solutions are used to provide a UWB infrastructure and a system and to work as location platform with a standard bidirectional time division multiple access control channel.
7	Pittet *et al.* [64]	2008	MEMS	AOA, TDOA		Combined UWB positioning with micro electro mechanical sensors (MEMS) inertial sensors in an extended Kalman filter to improve positioning and navigation performance.
8	Shahi *et al.* [47]	2012		AOA, TDOA	LOS, NLOS	Developed a UWB positioning system for material and activity tracking in indoor construction projects and studied the effect of construction materials on performance.
9	Segura *et al.* [48]	2012		TOA, TDOA	LOS, NLOS	Developed a UWB navigation system for mobile robot (MR) in indoor environments. Synchronization by the receivers is not required since a centralized transmitter with TDMA is used. Also, an adaptive threshold crossing algorithm is used to improve TOA estimator resistance to noise and interference.
10	Cao and Li [65]	2012		AOA, TOA	LOS	Developed a new $H_{\infty }$ filter based algorithm to estimate the location and velocity of a target object in a real-time.
11	Mucchi *et al.* [66]	2010		AOA, TOA	LOS	Developed an UWB real-time positioning system using Ubisense UWB solutions to provide cinematic data that helps in monitoring the performance of a professional athlete, especially after a surgery.
12	Liu *et al.* [67]	2012	GPS	TDOA	LOS, NLOS	Developed an indoor and outdoor cooperative real-time positioning system for disaster aid missions by considering their requirements.
13	Kuhn *et al.* [58]	2011	TDMA, FPGA	TDOA		Designed a multi-tag access scheme for UWB positioning systems with millimeter range accuracy for surgical navigation which allows simultaneous tracking of up to 30 UWB tags.
14	Zhang *et al.* [68]	2010	FPGA	TDOA, RSS		Presented a new noncoherent UWB indoor positioning system with millimeter range accuracy.
15	Deissler *et al.* [69]	2012	MIMO	AOA		Presented a new indoor mapping system using a UWB radar and a simple mobile antenna array with one transmitter and two receivers to extract the round-trip-times. In order to cope with lack of infrastructure and prior knowledge of the surrounding environment, a Rao-Blackwellized particle filter was used for estimation algorithms.
16	Tuchler *et al.* [70]	2005		TOA	LOS	Evaluated location accuracy of a UWB positioning system in an indoor environment showing that short transmitted pulses improve the accuracy in a multi-path environment.
17	Jiang *et al.* [71]	2012		TOA, RSS	LOS, NLOS	Proposed a new circuit to fully integrate a non-coherent IR-UWB transceiver, which rectified the baseband pulses and provided the digitized data to the digital baseband of the receiver.
18	Tomé *et al.* [72]	2010		TOA	LOS	Presented a large-scale deployable UWB-based indoor positioning system that relies on a developed application specific integrated circuits (ASICs) to provide a cost-competitive solution for possible future commercialization.
19	Arias-de-Reyna and Mengali [49]	2013		TOA	LOS	Built a new UWB indoor positioning system which relies on a combination of the maximum likelihood principle with range error models (and special fingerprints) based on prior knowledge obtained from the service area.
20	Kilic *et al.* [73]	2013		TOA	LOS	Proposed a new device-free stationary person detection and ranging method using existing fixed UWB infrastructure via detecting small low-frequency variations caused by a person’s presence.
21	Mahfouz *et al.* [74]	2011	FPGA	TDOA	LOS, NLOS	Designed a millimeter range UWB indoor positioning system for medical applications using an adaptive leading-edge detection algorithm to distinguish LOS from NLOS in order to optimize the ranging algorithm accordingly.
22	McCracken *et al.* [75]	2013	CIR	RSS	NLOS	Presented a device free positioning system that uses UWB radios together with RSS sensors to localize and track people through a building.
23	Jiang *et al.* [76]	2013		TOA, TDOA		Presented a fast three dimensional node UWB positioning system that uses a modified propagator method for time delay estimation and a 3D Chan algorithm for position determination.
24	Yang *et al.* [77]	2013		TDOA	LOS	Proposed a space-time Bayesian compressed sensing (STBCS) algorithm for the compressed sensing UWB positioning system to decrease the ADC sampling rate and improve noise tolerance.
25	Mirza *et al.* [78]	2012	Ultrasound sensor, compass	AOA, TDOA	LOS	Proposed a UWB indoor positioning and navigation system using Ubisense technologies to help physically disabled people to perform their daily activities.
26	Ubisense [79]	2010		AOA, TDOA	LOS, NLOS	Developed a UWB real-time vehicle tracking system to help dispatch managers in assigning vehicle to particular track or parking place at a yard area.
27	Ubisense [80]	2011		AOA, TDOA	LOS, NLOS	Developed a UWB real-time bus tracking system that manages buses parking and driver assignments in the company’s yard area using Ubisense technology.
28	Ubisense [81]	2010		AOA, TDOA	LOS, NLOS	Developed a UWB real-time positioning and tracking system for personnel safety in the oil and gas industry where devices and sensors cannot hold or produce enough energy to create sparks and should meet safety requirements.
29	Manon kok *et al.* [82]	2015	Inertial sensor	TOA	LOS, NLOS	Designed a novel approach for UWB calibration which consider the possibility of UWB delays due to NLOS and multipath.
30	Harikrishnan Ravikrishnan [83]	2014		AOA, TDOA	LOS	Used a map of an assembly line to improve UWB position tracking for indoor experiments with eight Ubisense sensors where inAve are located inside a laboratory and three outside.
31	Bharadwaj *et al.* [84]	2014	CIR, Peak detection	TOA	LOS, NLOS	Used UWB to locate body-worn sensors with different configurations and shapes.
32	Zaric *et al.* [85]	2013	Optical localization algorithm	TOA	LOS	Implemented a trilateration algorithm to calculate positions.
33	Ruiqing Ye [86]	2012		TDOA	LOS	Proposed new method to reduce the effect of path overlap and outperforms other methods such as first peak and search subtract and readjust (SSR) methods.
34	Zwirello *et al.* [87]	2012		TDOA, AOA	LOS	Combined TDOA and AOA in localization systems to achieve best results.
35	Wang and Zhang [88]	2014	Joint Estimation	TOA, AOA	NLOS	Used sparse representation framework for joint estimation of TOA and AOA.
36	Muller *et al.* [89]	2014		TOA	LOS, NLOS	Compared generalised Gaussian mixtures (GGM) with extended Kalman filter in nvironment with uncertainty.
37	Leitinger *et al.* [90]	2014		RSS	NLOS	Combined maximum likelihood estimator with floor plan information to improve accuracy.
38	Garcia *et al.* [91]	2015		TDOA	LOS, NLOS	Applied extended Kalman filter to improve accuracy in a highly complex indoor scenario.
39	Perrat *et al.* [92]	2015		TOA, TDOA	LOS	Used Ubisense for real-time positioning of wheelchair athletics.

**Table 8:** Comparison of UWB Algorithms.

Criteria	AOA	TOA	TDOA	RSS
Position Estimation	The intersection of several pairs of angle direction lines	Time taken by the signal to go from the target node to several reference nodes	The delta in time between the signal’s arrival at multiple reference nodes	The received signals strength from several reference nodes at the target node
		The distance is directly proportional to the propagation time	The time differences are mapped to multiple intersected hyperbolas	The distance is inversely proportional to the signal strength
2D space	At least two reference nodes	At least three reference nodes	At least three reference nodes	At least three reference nodes
3D space	At least three reference nodes	At least four reference nodes	At least four reference nodes	At least four reference nodes
Synchronization	Lower requirement in terms of clock precision and synchronization	All transmitters and receivers in the system have to be precisely synchronized	Only the reference nodes need to be synchronized	Not required
		Difficult and costly		
LOS vs NLOS	Require a clear line-of-sight (LOS) between sender and receiver			Prefer LOS to reduce multipath effects
	Multi-Path effects change phase of a signal and cause large position error			Great negatively affected by existence of obstacles and walls
Issues	Small errors in angle measurement will negatively impact accuracy	Relative clock drift between sender and receiver	Lower accuracy than TOA with the same system geometry	Sensitive to channel inconsistency
	Require costly and large dimensions of antenna arrays			Require short distances between nodes

**Table 9:** Summary of strengths, weaknesses, opportunities, and threats (SWOT) analysis for UWB technology.

**Internal Factors**	
**Strengths**	**Weaknesses**
License free Low power consumption Does not interfere with most of the existing radio systems High level of multipath resolution Large bandwidth High data rate communication High processing gain in communication system Involves very short pulses Carrierless transmission property offers the advantage of hardware simplicity. Works well with low SNR Low probability of interception and detection Resistance to jamming Can penetrate different kinds of materials	Potential interference to the existing systems which operates in the ultra wide spectrum due to misconfiguration (e.g., Wimax in the United States) May affect GPS and aircraft navigation radio equipment Very short pulses in UWB may take a long time to synchronize
**External Factors**	
**Opportunities**	**Threats**
Robot guidance Tracking systems Medical procedures and surgeries that require sub-millimeters of accuracy Indoor localization systems Short pulses which can be utilized for non-communication purposes Sensor, positioning, and identification network (SPIN) Industrial warehouse applications Shipboard environment applications Military applications Applications for noisy environments	Commercially expensive compared to other technologies In some cases not totally immune to multipath effects Design and implementation of UWB antennas can be more challenging

## Abbreviations

The following abbreviations are used in this manuscript:

**Definition**	**Acronyms**
Biased Kalman Filtering	BKF
Binary Phase Shift Keying	BPSK
Carrier Sense Multiple Access with Collision Avoidance	CSMA/CA
Channel Impulse Response	CIR
Extended Kalman Filter	EKF
Federal Communications Commission	FCC
Full Function Device	FFD
Global Positioning System	GPS
Global System for Mobile Communications	GSM
Impulse Radio Ultra WideBand	IR-UWB
Indoor Positioning System	IPS
Infrared	IR
Infrared Data Association	IrDA
Joint Committee for Guides in Metrology	JCGM
Least Square with Distance Contraction	LS-DC
Line of Sight	LOS
Local Area Network	LAN
Low data Rate UWB	LR-UWB
Maximum Likelihood Estimation	MLE
Micro Electro Mechanical Sensors	MEMS
Minimum-Shift Keying	MSK
Non-Line-of-Sight	NLoS
On-Off Keying	OOK
Pseudo Random	PR
Pulse Amplitude Modulation	PAM
Pulse Position Modulation	PPM
Pulse Width Modulation	PWM
Radio Frequency	RF
Radio-Frequency Identification	RFID
Real-Time Location System	RTLS
Received Signal Strength	RSS
Reduced Function Device	RFD
Strengths, Weaknesses, Opportunities, and Threats	SWOT
Time Difference of Arrival	TDOA
Time Division Multiple Access	TDMA
Time-Hopping Binary Phase Shift Keying	TH-BPSK
Time-Hopping Pulse Position Modulation	TH-PPM
Time-Hopping Spread Spectrum	TH-SS
Time Modulated Ultra WideBand	TM-UWB
Angle of Arrival	AOA
Time of Arrival	TOA
Transmit Power Control	TPC
Ultra WideBand	UWB
Weighted Least Square with Multidimensional Scaling	WLS-MDS
Wireless Fidelity	WiFi
Wireless Local Area Network	WLAN
Wireless Personal Area Network	WPAN
Worldwide Interoperability for Microwave Access	WiMAX

## References

[1] Hightower J., Borriello G. (2001). Location systems for ubiquitous computing. IEEE Comput..

[2] Huang H., Gartner G., Gartner G., Ortag F. Chapter 20, A Survey of Mobile Indoor Navigation Systems. Cartography in Central and Eastern Europe.

[3] Liu H., Darabi H., Banerjee P., Liu J. (2007). Survey of wireless indoor positioning techniques and systems. IEEE Trans. Syst. Man Cybern. Part C Appl. Rev..

[4] Ram S., Sharf J. The people sensor: A mobility aid for the visually impaired. Proceedings of the Second International Symposium on Wearable Computers.

[5] Gu Y., Lo A., Niemegeers I. (2009). A survey of indoor positioning systems for wireless personal networks. Tutor. IEEE Commun. Surv..

[6] Jekabsons G., Kairish V., Zuravlyov V. (2011). An Analysis of Wi-Fi Based Indoor Positioning Accuracy. Sci. J. Riga Tech. Univ. Comput. Sci..

[7] Wu H., Marshall A., Yu W. Path planning and following algorithms in an indoor navigation model for visually impaired. Proceedings of the Second International Conference on Internet Monitoring and Protection, ICIMP 2007.

[8] Al-Ammar M., Alhadhrami S., Al-Salman A., Alarifi A. Comparative Survey of Indoor Positioning Technologies, Techniques, and Algorithms. Proceedings of the 2014 International Conference on Cyberworlds (CW).

[9] Pandey S., Agrawal P. (2006). A survey on localization techniques for wireless networks. J. Chin. Inst. Eng..

[10] Khodjaev J., Park Y., Malik A. (2010). Survey of NLOS identification and error mitigation problems in UWB-based positioning algorithms for dense environments. Ann. Telecommun..

[11] Honkavirta V., Perala T., Ali-Loytty S., Piché R. A comparative survey of WLAN location fingerprinting methods. Proceedings of the 6th Workshop on Positioning, Navigation and Communication, WPNC 2009.

[12] Wang J., Ghosh R., Das S. (2010). A survey on sensor localization. J. Control Theory Appl..

[13] Guvenc I., Chong C. (2009). A survey on TOA based wireless localization and NLOS mitigation techniques. IEEE Commun. Surv. Tutor..

[14] Ruiz-López T., Garrido J., Benghazi K., Chung L. (2010). A Survey on Indoor Positioning Systems: Foreseeing a Quality Design. Distributed Computing and Artificial Intelligence.

[15] Al Nuaimi K., Kamel H. A survey of indoor positioning systems and algorithms. Proceedings of the 2011 International Conference on Innovations in Information Technology (IIT), IEEE Society.

[16] Ijaz F., Yang H., Ahmad A., Lee C. Indoor positioning: A review of indoor ultrasonic positioning systems. Proceedings of the 2013 15th International Conference on Advanced Communication Technology (ICACT).

[17] Adalja Disha M. (2013). A Comparative Analysis on Indoor Positioning Techniques and Systems. Int. J. Eng. Res. Appl. (IJERA).

[18] Zhu L., Yang A., Wu D., Liu L. (2014). Survey of Indoor Positioning Technologies and Systems. Life System Modeling and Simulation.

[19] Alhadhrami S., Al-Salman A., Al-Khalifa H., Alarifi A., Alnafessah A., Alsaleh M., Al-Ammar M. Ultra Wideband Positioning: An Analytical Study of Emerging Technologies. Proceedings of the Eighth International Conference on Sensor Technologies and Applications, SENSORCOMM 2014.

[20] Mautz R. (2012). Indoor Positioning Technologies. Ph.D. Thesis.

[21] Shi J.D. (2013). The Challenges of Indoor Positioning.

[22] Collin J., Mezentsev O., Lachapelle G. Indoor positioning system using accelerometry and high accuracy heading sensors. Proceedings of the ION GPS/GNSS 2003 Conference.

[23] Chóliz J., Eguizabal M., Hernandez-Solana A., Valdovinos A. Comparison of Algorithms for UWB Indoor Location and Tracking Systems. Proceedings of the 2011 IEEE 73rd Conference on Vehicular Technology Conference (VTC Spring).

[24] Beauregard S., Haas H. Pedestrian dead reckoning: A basis for personal positioning. Proceedings of the 3rd Workshop on Positioning, Navigation and Communication.

[25] Svalastog M.S. (2007). Indoor Positioning-Technologies, Services and Architectures. Cand Scient Thesis.

[26] Hightower J., Borriello G. (2001). Location Sensing Techniques.

[27] Song Z., Jiang G., Huang C. (2011). A Survey on Indoor Positioning Technologies. Theoretical and Mathematical Foundations of Computer Science.

[28] Gorostiza E.M., Lázaro Galilea J.L., Meca Meca F.J., Salido Monzú D., Espinosa Zapata F., Pallarés Puerto L. (2011). Infrared sensor system for mobile-robot positioning in intelligent spaces. Sensors.

[29] Brassart E., Pegard C., Mouaddib M. (2000). Localization Using Infrared Beacons. Robotica.

[30] Aitenbichler E., Muhlhauser M. An IR local positioning system for smart items and devices. Proceedings of the 23rd International Conference on Distributed Computing Systems Workshops.

[31] Rapinski J., Smieja M. (2015). ZigBee Ranging using Phase Shift Measurements. J. Navig..

[32] Rapinski J. (2015). The Application of ZigBee Phase Shift Measurement in Ranging. Acta Geodyn. Geomater..

[33] Ivanov R. Indoor navigation system for visually impaired. Proceedings of the 11th International Conference on Computer Systems and Technologies and Workshop for PhD Students in Computing on International Conference on Computer Systems and Technologies.

[34] Mautz R., Tilch S. Survey of optical indoor positioning systems. Proceedings of the 2011 International Conference on Indoor Positioning and Indoor Navigation (IPIN).

[35] Eriksson R. (2005). Indoor Navigation with Pseudolites (fake GPS sat.). Master’s Thesis.

[36] Wang J. (2002). Pseudolite applications in positioning and navigation: Progress and problems. J. Glob. Position. Syst..

[37] Barnes J., Rizos C., Wang J., Small D., Voigt G., Gambale N. Locata: A new positioning technology for high precision indoor and outdoor positioning. Proceedings of the 2003 International Symposium on GPS∖GNSS.

[38] Fluerasu A., Jardak N., Vervisch-Picois A., Samama N. (2011). Status of the GNSS transmitter-based approach for indoor positioning. Coord. Mag..

[39] Niwa H., Kodaka K., Sakamoto Y., Otake M., Kawaguchi S., Fujii K., Kanemori Y., Sugano S. GPS-based indoor positioning system with multi-channel pseudolite. Proceedings of the IEEE International Conference on Robotics and Automation, ICRA 2008.

[40] Alawieh M., Patino-Studencka L., Dahlhaus D. Stochastic modeling of pseudolite clock errors using enhanced AR methods. Proceedings of the 2010 7th International Symposium on Communication Systems Networks and Digital Signal Processing (CSNDSP).

[41] Bowdith N. (2002). Chapter 7, The American Practical Navigator. Part F: Robotics.

[42] Liu J., Shi D., Leung K. Indoor navigation system based on omni-directional corridorguidelines. Proceedings of the 2008 International Conference on Machine Learning and Cybernetics.

[43] Babu R., Wang J. Ultra-tight integration of pseudolites with INS. Proceedings of the 2006 IEEE/ION Position, Location, and Navigation Symposium.

[44] Ghavami M., Michael L.B., Kohno R. (2006). Front MatterUltra Wideband Signals and Systems in Communication Engineering.

[45] Siwiak K., McKeown D. (2005). Ultra-Wideband Radio Technology.

[46] Cheng G. (2012). Accurate TOA-based UWB localization system in coal mine based on WSN. Phys. Proced..

[47] Shahi A., Aryan A., West J., Haas C., Haas R. (2012). Deterioration of UWB positioning during construction. Autom. Constr..

[48] Segura M., Mut V., Sisterna C. (2012). Ultra wideband indoor navigation system. IET Radar Sonar Navig..

[49] Arias-de Reyna E., Mengali U. (2013). A maximum likelihood UWB localization algorithm exploiting knowledge of the service area layout. Wirel. Pers. Commun..

[50] Krishnan S., Sharma P., Guoping Z., Woon O. A UWB based localization system for indoor robot navigation. Proceedings of the IEEE International Conference on Ultra-Wideband, ICUWB 2007.

[51] Ubisense Company Ubisense Website, 2009. http://www.ubisense.net/en/.

[52] Technavio Global Indoor LBS Market 2015–2019. http://www.technavio.com/report/global-indoor-lbs-market-2015-2019.

[53] Alereon Inc. (2015). Alereon Demonstrates Military Wireless Personal Area Network. http://www.alereon.com/?page_id=2992.

[54] Phebey T. (2010). The Ubisense Assembly Control Solution for BMW. https://scholar.google.com/scholar?hl=en&q=ubisense+BMW&btnG=&as_sdt=1%2C5&as_sdtp=.

[55] Gabriel C. (2014). UWB’s Dream is Still Alive in Micro-Location. http://www.rethink-wireless.com/2014/10/21/uwbs-dream-alive-micro-location-page1.

[56] Krulwich B. Ultra-Wideband Poised to Enter Smartphones: A Location Opportunity. http://www.gpsbusinessnews.com/Ultra-Wideband-Poised-to-Enter-Smartphones-a-Location-Opportunity_a4969.html.

[57] Cui S. (2011). Modulation and Multiple Access Techniques for Ultra-Wideband Communication Systems. Ph.D. Thesis.

[58] Kuhn M., Mahfouz M., Turnmire J., Wang Y., Fathy A. A multi-tag access scheme for indoor UWB localization systems used in medical environments. Proceedings of the 2011 IEEE Topical Conference on Biomedical Wireless Technologies, Networks, and Sensing Systems (BioWireleSS).

[59] Davis M. (2011). Foliage Penetration Radar.

[60] Davis M.E. (2013). Frequency allocation challenges for ultra-wideband radars. IEEE Aerosp. Electron. Syst. Mag..

[61] Fischer G., Klymenko O., Martynenko D., Luediger H. An impulse radio UWB transceiver with high-precision TOA measurement unit. Proceedings of the 2010 International Conference on Indoor Positioning and Indoor Navigation (IPIN).

[62] Rowe N., Fathy A., Kuhn M., Mahfouz M. A UWB transmit-only based scheme for multi-tag support in a millimeter accuracy localization system. Proceedings of the 2013 IEEE Topical Conference on Wireless Sensors and Sensor Networks (WiSNet).

[63] Jiang L., Hoe L., Loon L. Integrated UWB and GPS location sensing system in hospital environment. Proceedings of the 2010 the 5th IEEE Conference on Industrial Electronics and Applications (ICIEA).

[64] Pittet S., Renaudin V., Merminod B., Kasser M. (2008). UWB and MEMS based indoor navigation. J. Navig..

[65] Cao F., Li M. (2012). An Algorithm for UWB Signals Tracking Based on Extended H Filter. Phys. Proced..

[66] Mucchi L., Trippi F., Carpini A. Ultra Wide Band real-time location system for cinematic survey in sports. Proceedings of the 3rd International Symposium on Applied Sciences in Biomedical and Communication Technologies (ISABEL).

[67] Liu J., Wang Q., Xiong J., Huang W., Peng H. (2012). Indoor and Outdoor Coperative Real-Time Positioning System. J. Theor. Appl. Inf. Technol. (JATIT).

[68] Zhang C., Kuhn M., Merkl B., Fathy A., Mahfouz M. (2010). Realtime non-coherent UWB positioning radar with millimeter range accuracy: Theory and experiment. IEEE Trans. Microw. Theory Tech..

[69] Deissler T., Janson M., Zetik R., Thielecke J. Infrastructureless indoor mapping using a mobile antenna array. Proceedings of the 2012 19th International Conference on Systems, Signals and Image Processing (IWSSIP).

[70] Tuchler M., Schwarz V., Huber A. Location accuracy of an UWB localization system in a multi-path environment. Proceedings of the 2005 IEEE International Conference on Ultra-Wideband, ICU 2005.

[71] Jiang X., Zhang H., Wang W. (2012). NLOS error mitigation with information fusion algorithm for UWB ranging systems. J. China Univ. Posts Telecommun..

[72] Tomé P., Robert C., Merz R., Botteron C. UWB-based Local Positioning System: From a small-scale experimental platform to a large-scale deployable system. Proceedings of the 2010 International Conference on Indoor Positioning and Indoor Navigation (IPIN).

[73] Kilic Y., Wymeersch H., Meijerink A., Bentum M., Scanlon W. (2013). UWB device-free person detection and localization. CoRR.

[74] Mahfouz M., Kuhn M., Wang Y., Turnmire J., Fathy A. Towards sub-millimeter accuracy in UWB positioning for indoor medical environments. Proceedings of the 2011 IEEE Topical Conference on Biomedical Wireless Technologies, Networks, and Sensing Systems (BioWireleSS).

[75] McCracken M., Bocca M., Patwari N. Joint ultra-wideband and signal strength-based through-building tracking for tactical operations. Proceedings of the 2013 10th Annual IEEE Communications Society Conference on Sensor, Mesh and Ad Hoc Communications and Networks (SECON).

[76] Jiang H., Zhang Y., Cui H., Liu C. Fast three-dimensional node localization in UWB wireless sensor network using propagator method digest of technical papers. Proceedings of the 2013 IEEE International Conference on Consumer Electronics (ICCE).

[77] Yang D., Li H., Zhang Z., Peterson G. (2012). Compressive sensing based sub-mm accuracy UWB positioning systems: A space–time approach. Digit. Signal Process..

[78] Mirza R., Tehseen A., Kumar A. An indoor navigation approach to aid the physically disabled people. Proceedings of the 2012 International Conference on Computing, Electronics and Electrical Technologies (ICCEET).

[79] Brown C. Real-Time Location of Jena’s Buses and Trams with Ubisense RTLS, 2010. Ubisense Report. http://www.ubisense.net/en/news-and-events/press-releases/real-time-location-of-jenas-buses-and-trams-with-ubisense-rtls.html.

[80] Baum M. (2011). RTL in Longueuil selects bus yard management solution provided by Solotech, ISR Transit and Ubisense. http://www.ubisense.net/en/news-and-events/press-releases/rtl-in-longueuil-selects-bus-yard.html.

[81] Brown C. (2010). Ubisense launches Intrinsically Safe location tracking tags for personnel safety in the Oil and Gas industry. http://www.ubisense.net/en/news-and-events/press-releases/ubisense-launches-intrinsically-safe-location-tracking-tags-in-the-oil-and-gas-industry.html.

[82] Kok M., Hol J.D., Schon T.B. (2015). Indoor positioning using ultrawideband and inertial measurements. IEEE Trans. Veh. Technol..

[83] Ravikrishnan H. (2014). Ultra-Wideband Position Tracking on an Assembly Line. Ph.D. Thesis.

[84] Bharadwaj R., Swaisaenyakorn S., Parini C.G., Batchelor J., Alomainy A. (2014). Localization of wearable ultrawideband antennas for motion capture applications. IEEE Antennas Wirel. Propag. Lett..

[85] Zaric A., Matos V.S., Costa J.R., Fernandes C.A. (2013). Viability of wall-embedded tag antenna for ultra-wideband real-time suitcase localisation. IET Microw. Antennas Propag..

[86] Ye R. (2012). Ultra-wideband Indoor Localization Systems. Ph.D. Thesis.

[87] Zwirello L., Schipper T., Harter M., Zwick T. (2012). UWB localization system for indoor applications: Concept, realization and analysis. J. Electric. Comput. Eng..

[88] Wang F., Zhang X. (2014). Joint estimation of TOA and DOA in IR-UWB system using sparse representation framework. ETRI J..

[89] Muller P., Wymeersch H., Piche R. (2014). UWB Positioning with Generalized Gaussian Mixture Filters. IEEE Trans. Mob. Comput..

[90] Leitinger E., Fröhle M., Meissner P., Witrisal K. Multipath-assisted maximum-likelihood indoor positioning using UWB signals. Proceedings of the 2014 IEEE International Conference on Communications Workshops (ICC).

[91] Garcia E., Poudereux P., Hernandez A., Urena J., Gualda D. A robust UWB indoor positioning system for highly complex environments. Proceedings of the 2015 IEEE International Conference on Industrial Technology (ICIT).

[92] Perrat B., Smith M.J., Mason B.S., Rhodes J.M., Goosey-Tolfrey V.L. (2015). Quality assessment of an UWB positioning system for indoor wheelchair court sports. SAGE J..

[93] Al-Jazzar S., Muchkaev A., Al-Nimrat A., Smadi M. (2011). Low complexity and high accuracy angle of arrival estimation using eigenvalue decomposition with extension to 2D AOA and power estimation. EURASIP J. Wirel. Commun. Netw..

[94] Reddy N., Sujatha B. (2011). TDOA Computation Using Multicarrier Modulation for Sensor Networks. Int. J. Comput. Sci. Commun. Netw..

[95] Gezici S. (2005). Localization via ultra-wideband radios: A look at positioning aspects for future sensor networks. IEEE Signal Process. Mag..

[96] Xu J., Ma M., Law C. AOA Cooperative Position Localization. Proceedings of the Global Telecommunications Conference, IEEE GLOBECOM 2008, IEEE.

[97] Lee Y. (2011). Weighted-Average Based AOA Parameter Estimations for LR-UWB Wireless Positioning System. IEICE Trans. Commun..

[98] Subramanian A. UWB Linear Quadratic Frequency Domain Frequency Invariant Beamforming and Angle of Arrival Estimation. Proceedings of the IEEE 65th Vehicular Technology Conference, VTC2007-Spring.

[99] Mok E., Xia L., Retscher G., Tian H. (2010). A case study on the feasibility and performance of an UWB-AoA real time location system for resources management of civil construction projects. J. Appl. Geod..

[100] Gerok W., El-Hadidy M., El Din S., Kaiser T. Influence of the real UWB antennas on the AoA estimation based on the TDoA localization technique. Proceedings of the 2010 IEEE Middle East Conference on Antennas and Propagation (MECAP).

[101] Dardari D., Conti A., Ferner U., Giorgetti A., Win M.Z. (2009). Ranging with ultrawide bandwidth signals in multipath environments. IEEE Proc..

[102] Blankenbach J., Norrdine A., Hellmers H. A robust and precise 3D indoor positioning system for harsh environments. Proceedings of the 2012 International Conference on Indoor Positioning and Indoor Navigation (IPIN).

[103] Leitinger E., Meissner P., Rudisser C., Dumphart G., Witrisal K. (2015). Evaluation of Position-Related Information in Multipath Components for Indoor Positioning. IEEE J. Sel. Areas Commun..

[104] Cyganski D., Orr J., Michalson W.R. A multi-carrier technique for precision geolocation for indoor/multipath environments. Proceedings of the 16th International Technical Meeting of the Satellite Division of The Institute of Navigation.

[105] Cyganski D., Orr J., Michalson W.R. Performance of a Precision Indoor Positioning System Using Multi Carrier Approach. Proceedings of the 2004 National Technical Meeting of The Institute of Navigation.

[106] Wang S., Waadt A., Burnic A., Xu D. System implementation study on RSSI based positioning in UWB networks. Proceedings of the 2010 7th International Symposium on Wireless Communication Systems (ISWCS).

[107] Gigl T., Janssen G., Dizdarevic V., Witrisal K., Irahhauten Z. Analysis of a UWB Indoor Positioning System Based on Received Signal Strength. Proceedings of the 4th Workshop on Positioning, Navigation and Communication, WPNC ’07.

[108] Kodippili N., Dias D. Integration of fingerprinting and trilateration techniques for improved indoor localization. Proceedings of the 2010 Seventh International Conference On Wireless And Optical Communications Networks (WOCN).

[109] Wymeersch H., Lien J., Win M.Z. (2009). Cooperative localization in wireless networks. IEEE Proc..

[110] Cao F., Li M. (2012). An Algorithm for UWB Signals Tracking Based on Extended H Filter. Phys. Proced..

[111] Liu J., Wang Q., Xiong J., Huang W., Peng H. (2013). Indoor and Outdoor Cooperative Real-Time Positioning System. J. Theor. Appl. Inf. Technol..

[112] Digel J., Masini M., Grozing M., Berroth M., Fischer G., Olonbayar S., Gustat H., Scheytt J.C. Integrator and Digitizer for a non-coherent IR-UWB Receiver. Proceedings of the 2013 IEEE 13th Topical Meeting on Silicon Monolithic Integrated Circuits in RF Systems (SiRF).

[113] Srimathi S., Kannan P. (2013). Literature survey for performance evaluation of various time hopping ultra-wideband communication system. Int. J. Sci. Eng. Res..

[114] Shen G., Zetik R., Thoma R. Performance comparison of TOA and TDOA based location estimation algorithms in LOS environment. Proceedings of the 5th Workshop on Positioning, Navigation and Communication (WPNC).

[115] Mallat A., Louveaux J., Vandendorpe L. UWB based positioning: Cramer Rao bound for Angle Of Arrival and comparison with Time Of Arrival. Proceedings of the 2006 Symposium on Communications and Vehicular Technology.

[116] Kułakowski P., Vales-Alonso J., Egea-López E., Ludwin W., García-Haro J. (2010). Angle-of-arrival localization based on antenna arrays for wireless sensor networks. Comput. Electr. Eng..

[117] Hatami A., Pahlavan K. Performance Comparison of RSS and TOA Indoor Geolocation Based on UWB Measurement of Channel Characteristics. Proceedings of the IEEE 17th International Symposium on Personal, Indoor and Mobile Radio Communications.

[118] Cong L., Zhuang W. (2002). Hybrid TDOA/AOA mobile user location for wideband CDMA cellular systems. IEEE Trans. Wirel. Commun..

[119] Zhao Y. (2002). Standardization of mobile phone positioning for 3G systems. IEEE Commun. Mag..

[120] Yan J. (2010). Algorithms for Indoor Positioning Systems Using Ultra-Wideband Signals.

[121] Hämäläinen M., Hovinen V., Latva-aho M. (1999). Survey to Ultra Wideband Systems. Eur. Cooperation Field Sci. Tech. Res..

[122] Otis B., Rabaey J. (2007). Ultra-Low Power Wireless Technologies for Sensor Networks.

[123] Aiello R., Batra A. (2006). Ultra Wideband Systems: Technologies and Applications.

[124] Miller L. (2003). Why UWB? a review of ultrawideband technology.

[125] Savioli A., Goldoni E., Gamba P. Impact of channel access on localization in cooperative UWB sensor network: A case study. Proceedings of the 2012 9th Workshop on Positioning Navigation and Communication (WPNC).

[126] Ye S., Chen J., Liu L., Zhang C., Fang G. A novel compact UWB ground penetrating radar system. Proceedings of the 2012 14th International Conference on Ground Penetrating Radar (GPR).

[127] Porcino D., Hirt W. (2003). Ultra-wideband radio technology: potential and challenges ahead. IEEE Commun. Mag..

